# An improved poor and rich optimization algorithm

**DOI:** 10.1371/journal.pone.0267633

**Published:** 2023-02-09

**Authors:** Yanjiao Wang, Shengnan Zhou

**Affiliations:** Department of Electrical Engineering, Northeast Electric Power University, Jilin, China; University of Konya Technical, TURKEY

## Abstract

The poor and rich optimization algorithm (PRO) is a new bio-inspired meta-heuristic algorithm based on the behavior of the poor and the rich. PRO suffers from low convergence speed and premature convergence, and easily traps in the local optimum, when solving very complex function optimization problems. To overcome these limitations, this study proposes an improved poor and rich optimization (IPRO) algorithm. First, to meet the requirements of convergence speed and swarm diversity requirements across different evolutionary stages of the algorithm, the population is dynamically divided into the poor and rich sub-population. Second, for the rich sub-population, this study designs a novel individual updating mechanism that learns from the evolution information of the global optimum individual and that of the poor sub-population simultaneously, to further accelerate convergence speed and minimize swarm diversity loss. Third, for the poor sub-population, this study designs a novel individual updating mechanism that improves some evolution information by learning alternately from the rich and Gauss distribution, gradually improves evolutionary genes, and maintains swarm diversity. The IPRO is then compared with four state-of-the-art swarm evolutionary algorithms with various characteristics on the CEC 2013 test suite. Experimental results demonstrate the competitive advantages of IPRO in convergence precision and speed when solving function optimization problems.

## 1. Introduction

Swarm intelligence evolutionary algorithms are meta-heuristic optimization algorithms inspired by the natural swarm intelligence phenomenon. These algorithms can obtain the optimal solution without the need to be continuously differentiable in the search space when solving optimization problems, and are robustness, simplicity and extensibility, among others. Given these characteristic, swarm intelligence evolutionary algorithms have been widely used in various engineering fields, such as community detection for complex networks [1], wireless sensor networks [2], pattern recognition [3], parameter estimation [4], image processing [5], and target tracking [6], *etc*. To further improve the application effectiveness of these algorithms in engineering, scholars have attempted to further improve their optimization performance by designing many new swarm intelligence algorithms, and improving the performance of existing algorithms.

At present, many swarm intelligence evolutionary algorithms have been proposed, and can be roughly divided into three categories according to the heuristic principle. First The swarm heuristic algorithms are proposed by simulating the living habits of organisms in nature, such as particle swarm optimization (PSO) algorithm [7], artificial fish swarm algorithm(AFSA) [8], ant colony optimization(ACO) algorithm [9], bacterial foraging algorithm(BFA) [10], artificial bee colony algorithm (ABC) [11], glowworm swarm optimization algorithm(GSO) [12], firefly algorithm (FA) [13], cat swarm optimization (CSO) [14], cockroach swarm algorithm(CSA) [15], monkey algorithm (MA) [16], bat algorithm(BA) [17], zombie survival optimization(ZSO) [18], krill herd (KH) algorithm [19], migrating birds optimization(MBO) algorithm [20], dolphin echolocation(DE) algorithm [21], social spider optimization (SSO) algorithm [22], gray wolf optimization(GWO) algorithm [23], chicken swarm optimization (CSO) algorithm [24], moth flame optimization(MFO) algorithm [25], monarch butterfly optimization (MBO) algorithm [26], dragonfly algorithm(DA) algorithm [27], dolphin swarm algorithm (DSA) algorithm [28], elephant swarm water search algorithm(ESWS) [29], whale optimization algorithm(WOA) [30], circular structures of puffer fish algorithm(CSOPF) [31], and poor and rich optimization algorithm(PRO) [32], etc. Second, The swarm intelligence evolutionary algorithms are inspired by some physical phenomena. For instance, Birbil *et al*. proposed electromagnetism-like meta-heuristic (EM) algorithm [33], Menser *et al*. proposed particle swirl algorithm(PSA) inspired by vortex motion [34], Erol *et al*. proposed big bang-big crunch(BB-BC) algorithm according to big bang theory and contraction theory [35], Rashedi *et al*. proposed gravitational search algorithm (GSA) based on the Law of Universal Gravitation proposed by Newton [36], Ying Tan *et al*. proposed firework algorithm (FWA) by simulating the explosion and lighting process of real fireworks in the night sky [37], A. Kaveh *et al*. proposed water evaporation optimization (WEO) algorithm [38] inspired by the evaporation process of a few molecules on solid surfaces with different wettability, Javidy et al. proposed ion motion optimization (IMO) algorithm according to the law of motation and transformation of particles in liquid state and solid state [39], Fatma A. Hashim et al. proposed Archimedes optimization algorithm (AOA) [40], which is devised with inspirations from an interesting law of physics Archimedes’ Principle. Third, swarm intelligence evolutionary algorithms are inspired by the genetic evolution process, such as the famous GA, differential evolution (DE), clone selection algorithm (CSA) inspired by clone selection mechanism, immune algorithm (IA), social cognitive optimization(SCO) [41], free search (FS) algorithm [42], harmony search (HS) algorithm [43], Biogeography-Based Optimization (BBO) algorithm [44] proposed by Simon et al. IEEE Transaction on Evolutionary Computation, brain storm optimization(BSO), teaching-learning-based optimization(TLBO) algorithm [45], symbiotic organisms search (SOS) algorithm [46], animal migration optimization (AMO) algorithm [47], *etc*.

To further improve the performance of various swarm intelligence evolutionary algorithms, scholars have proposed many improved evolutionary algorithms. For instance, Wei Sun *et al*. proposed an improved particle swarm optimization algorithm based on an all-dimension-neighborhood-based PSO with the randomly selected neighbors learning strategy (AND-RSN-PSO) [48]. At the early stage of PSO, they adopted the randomly selected neighbors (RSN) learning strategy to enhance swarm diversity, whereas at the later stage, they used the all-dimension neighborhood (ADN) strategy to accelerate the convergence rate. Meng Wang *et al*. proposed a modified sine cosine algorithm (MSCA) [49], which introduces linear searching path and empirical parameter to improve the search path of original SCA. Deng xianli *et al*. proposed a multi-population based self-adaptive migration PSO (MSMPSO) [50], which integrates the two common neighbor typologies into particle’s social-learning part. Mahmoud M. Saafan *et al*. proposed a hybrid improved whale optimization salp swarm algorithm (IWOSSA) [51], which combines improved whale optimization algorithm and salp swarm algorithm. Yaping xiao *et al*. designed a variety of efficient update operators and redesigned the update strategies of branches, and proposed an improved artificial tree algorithm with two populations (IATTP) [52]. Rehab ali Ibrahim *et al*. proposed an improved version of the gray wolf optimizer, named chaotic opposition-based Grey-Wolf optimization algorithm based on differential evolution and disruption optimization (COGWO2D) [53]. Seyed Mostafa Bozorgi *et al*. proposed an improved whale optimization algorithm (REWOA), which combines exploitation of WOA with exploration of DE and therefore provides a promising candidate solution [54]. Omid Trakhaneh *et al*. proposed an improved differential evolution algorithm using Archimedean Spiral and neighborhood search based mutation approach (ADENS) [55]. Emine Bas *et al*. added two new techniques to the original social spider algorithm to propose an improved social spider algorithm (ISSA) [56].

Among the above swarm intelligence algorithms, the poor and rich optimization algorithm (PRO) was recently proposed by V.K. Bardsiri *et al*., by taking inspiration from the behavior of the poor and the rich in acquiring wealth to improve their economic conditions. Many experiments have confirmed that PRO has significantly better optimization performance than PSO, ABC, and SCA, *etc*. It suggests that PRO also shows excellent application potential in various engineering fields. Unfortunately, similar to other swarm intelligence evolutionary algorithms, PRO demonstrates low convergence speed and precision when solving very complex optimization problems, and no study thus far has attempted to improve the performance. To improve the effectiveness of PRO in engineering application, this study designs an improved PRO (IPRO) algorithm. The strong motivation and main contributions of this paper are as follows. First, the population is divided into the poor and rich sub-population dynamically to replace the original fixed allocation model, in order to meet the convergence speed and population diversity requirements across different evolutionary stages. Second, a novel individual updating method for the rich is proposed to further accelerate the convergence speed and prevent excessive losses in population diversity. In this method, the rich learn from the poor and the top best individuals, and the gradually increases proportion of learning from the poor as the iteration progresses. Third, a novel individual updating method for the poor is designed to obtain reliable evolutionary information and maintain population diversity. Here, a new poor is obtain by retaining itself and a offspring individual according to some cross probability, where a offspring individual is obtained by the alternating updating model of Gaussian mutation and learning from the rich individuals. Results from the CEC2013 test suite show that the IPRO significantly outperforms four state-of-the-art optimization algorithms in terms of convergence precision and speed when solving function optimization problems.

The rest structure of this paper is organized as follows: Section 2 introduces the preliminaries, including the related introduction of optimization approaches and terminology, and the principle of the original PRO algorithm; Section 3 describes the innovation, the principle, procedures, and detailed operations of our proposed IPRO algorithm; in section 4, The proposed algorithm is tested and the results are analyzed. Finally, summarize the full paper.

## 2. Preliminaries

### 2.1 Terminology

The unconstrained optimization problem can be represented mathematically as

minimize  F=f(X) (1)

where ***X*** is a vector that represents an independent variable set, and ***F*** represents the objective function to be optimized.

It is noted that, this paper only researches on employing the swarm intelligence optimization algorithms to solve the above unconstrained optimization problem. The following terminology will be used in this paper.

**Chromosome:** Chromosomes can also be called individuals. A certain number of individuals form a population, and the number of individuals in the population is called population size.

**Gene:** Genes are elements in a string. Genes are used to represent individual characteristics. For example, if there is a string S = 1 0 1 1, the four elements 1, 0, 1 and 1 are called genes respectively. Their values are called alleles.

**Gene locus:** Gene locus represents the position of a gene in the string in the algorithm, which is also called gene position. The gene position is calculated from the left to the right of the string. For example, in a string S = 1 0 1 1, the gene position of 0 is 3.

**Fitness:** The degree of adaption of each individual to the environment is called fitness. In order to reflect the adaptability of chromosomes, a function that can measure each chromosome in the problem is introduced, which is called fitness function.

### 2.2 Poor and rich optimization algorithm

According to their wealth, people in society can be divided into the poor and the rich, and these two groups try to improve their economic situations in different ways. V.K. Bardsiri *et al*. simulating such behavior in 2019 to design the poor and rich optimization algorithm (PRO) [32]. In this algorithm, people are equivalent to individuals, and their economic status is equivalent to their fitness values. The poor improves its economic situation by narrowing its economic gap with the rich, whereas the rich increases its economic advantage by increasing its economic gap with the poor. The economic status of the entire population can be improved effectively by improving both the poor and the rich.

The pseudo code of PRO is shown in Algorithm 1. The detailed procedure of the original PRO algorithm is described as follows. First, initialize various parameters including *N*, *D*, *U*, *L*, *T*, and Pmut. Where N represents the number of individuals, D represents the dimension of the optimization problem, U and L denote the upper and lower limits of the search range respectively, *T* denotes the maximum number of iterations, and Pmut denotes the mutation probability. Afterwards, randomly generate the initial population *X*, and calculate the fitness value of each individual *X*(*i*). Second, the population X is divided into two sub-population according to fitness value as follows. The half of the individuals with better fitness value in *X* consist of the rich sub-population *X*_*rich*, and the others in *X* consist of the poor sub-population *X*_*poor*. Third, update each individuals of *X*_*rich* according to Eq (2) to form an new rich population *X*_*rich*^*new*^. Then update *X*_*rich*^*new*^ according to the following mutation operation. For each individual *X*_*rich*^*new*^ (*i*), generate a random number (i.e. *rand*). If *rand is* smaller than Pmut, then *X*_*rich*^*new*^ (*i*) will be replaced by *X*_*rich*^*new*^ (*i*) which adds a value of a normal distribution with average of 0 and variance of 1. Afterwards, compare *X*_*rich*^*new*^ (*i*) with *X*(*i*) as follows. If *X*_*rich*^*new*^ (*i*) is better than *X*(*i*), then *X*(*i*) will be replaced by *X*_*rich*^*new*^ (*i*). Fourth, update each individuals of *X*_*poor* according to Eq (3) to form the new rich population *X*_*poor*^*new*^. Then update *X*_*poor*^*new*^ according to the following mutation operation. Fifth, combine *X*_*rich*^*new*^ and *X*_*poor* to form X, and find the best individual of X. If the number of iteration reaches the maximum number of iteration, then the algorithm terminates. Otherwise, go to the second step for another round of iteration.

**Algorithm 1** PRO

**Input**: *N*, *D*, *U*, *L*, *T*, Pmut

**Output:** Best solution *x*_*best*_ and its fitness

01 Parameter initialization (*N*, *D*, *U*, *L*, *T*, *Pmut*)

02 *X*←Generate the initial population at random

03 *f* (*X*_*i*_), *i* ∈ (1,*N*) ← Calculate the fitness value of each individual

04 *t = 0*

05  **While** *t< = T do*

06   The population X is divided into two subpopulation according to fitness value, including the rich population *X*_*rich* and the poor population *X*_*poor*

07   *X*_*rich*^*new*^ ← Perform the individual updating method on *X*_*rich* according to Section 2.1

08   *X*_*rich*^*new*^ ← Perform the following mutation operation on *X*_*rich*^*new*^

  **for** i = 1:N/2

   **if** rand < Pmut

    *X*_*rich*^*new*^(*i*) = *X*_*rich*^*new*^ (*i*) + *randn*, // *randn* is the value of a normal distribution with a mean of 0 and a variance of 1

   **end if**

   **if** *f*(*X*_*rich*^*new*^(*i*)) > *f*(*X*_*rich*(*i*))

    *X*_*rich*^*new*^(*i*) = *X*_*rich*(*i*)

   **end If**

  **end For**

09  *X*_*poor*^*new*^ ← Perform the individual updating method on *X*_*poor* according to Section 2.2

10  *X*_*poor*^*new*^ ← Perform the mutation operation on *X*_*poor*^*new*^

11  *X* ← *X*_*rich*^*new*^ ∪ *X*_*poor*^*new*^

12  *x*_*best*_ ← best(X) // assign current best

13  *t = t+*1

14 **end While**

15 Return *x*_*best*_

#### 2.2.1 Individual updating method for the rich

Each individual in the rich sub-population is updated as

$$X\_rich^{new}(i)=X\_rich^{old}(i)+r\times (X\_rich^{old}(i)-X\_poor^{old}(best))$$ (2)

where *X*_*rich*^*new*^(*i*) and *X*_*rich*^*old*^(*i*) represent the *i*-th new individual and the *i*-th individual in the new rich sub-population and original rich sub-population, respectively, r is a random number between 0 and 1, and *X*_*poor*^*old*^(*best*) is the individual with the best fitness value in the poor sub-population.

#### 2.2.2 Individual updating method for the poor

Each individual in the poor sub-population is updated as

$$X\_poor^{new}(i)=X\_poor^{old}(i)+(r\times Pattern-X\_poor^{old}(i))$$ (3)

where *X*_*poor*^*old*^(*i*) and *X*_*poor*^*new*^(*i*) represent the *i*-th new individual and *i*-th individual in the new and original poor sub-population, respectively, *Pattern* is calculated as

$$Pattern=\frac{X\_rich^{old}(best)+X\_rich^{old}(mean)+X\_rich^{old}(worst)}{3}$$ (4)

where *X*_*rich*^*old*^(*best*), *X*_*rich*^*old*^(*worst*) and *X*_*rich*^*old*^(*mean*) represent the individual with the best fitness value, the individual with the worst fitness value, and the mean of all individuals in the rich sub-population, respectively.

## 3. Proposed algorithm

Reference [32] confirmed in their experiments that PRO can obtain the optimum solution for less complex function optimization problems. Unfortunately, for the highly complex optimization problems, PRO has slow convergence speed and easily falls into local optimal. To address these issues, this paper designs an improved poor and rich (IPRO)algorithm, whose pseudo code is presented in Algorithm 2.

**Algorithm 2**:IPRO

**Input**: *N*, *D*, *U*, *L*, *MaxFEs*

**Output:** *x*_*best*_, *f*(*x*_*best*_) //best solution and its fitness value

01 Parameter initialization (*N*, *D*, *U*, *L*, *MaxFEs*, *Pmut*)

02 *X* ← Generate the initial population randomly

03 *f*(*X*(*i*)), *i* ∈ (1,*N*) ← Calculate the fitness value of each individual *X*(*i*) in *X*

04 *FEs = 0*

05 **While** *FEs <= MaxFEs do*

06  *X*_*rich*, *X*_*poor* ← perform dynamic population division method on *X a*ccording to Section 3.1

07  *X*_*rich*^*new*^ ← perform the individual updating method on *X*_*rich a*ccording to Section 3.2

08  *X*_*poor*^*new*^ ← perform the individual updating method on *X*_*poor a*ccording to Section 3.3

09  *X*^*new*^ ← *X*_*rich*^*new*^ ∪ *X*_*poor*^*new*^

10  *X* ← perform the mutation operation similar to the method of Algorithm 1 on *X*^*new*^

   **for** *i* = 1 to *N*

    **if** rand < Pmut

     *X*^*new*^(*i*) = *X*^*new*^(*i*) + *randn*

    **end if**

    **if** *f*(*X*^*new*^(*i*)) < *f*(*X*(*i*))

     *X*(*i*) = *X*^*new*^ (*i*)

    **end If**

   **end for**

11  *x*_*best*_ ← best(X) // assign current best

12  update *FEs*

13 **End While**

14 return *x*_*best*_ and its fitness value *f*(*x*_*best*_)

The detailed step-by-step procedure for Algorithm 2 is described as follows:

**Step 1**: Initialize various parameters including *N*, *D*, *U*, *L*, *MaxFEs*, and Pmut. The parameters N, *D*, *U*, *L*, and Pmut are similar to those used in Algorithm 1, whereas *MaxFes* denotes the maximum number of function evaluations.
**Step 2**: Randomly generate the initial population *X*, and calculate the fitness value of each individual in *X*.
**Step 3**: Determine the size of the rich sub-population *X*_*rich* and the poor sub-population *X*_*poor* according to Eq (5) (i.e. |*X*_*rich*| = *z* and |*X*_*poor*| = *N*–*z*). Select the top z best individuals of *X* to form *X*_*rich*, and the others in *X* consist of *X*_*poor*. Further details are presented in Section 3.1.
**Step 4**: Apply the individual updating method on *X*_*rich* and *X*_*poor* as described in Sections 3.2 and 3.3, respectively, to form the new rich sub-population *X*_*rich*^*new*^ and the new poor sub-population *X*_*poor*^*new*^. Afterward, combine *X*_*rich*^*new*^ and *X*_*poor*^*new*^ to form a new population *X*^*new*^.
**Step 5**: Perform the mutation operation on *X*^*new*^ according to the following method. For each individual *X*^*new*^ (*i*), generate a random number (i.e. *rand*). If *rand is* smaller than Pmut, then *X*^*new*^ (*i*, *j*) will be replaced by *X*^*new*^ (*i*, *j*), which adds a value of normal distribution with an average of 0 and variance of 1. Afterward, compare *X*^*new*^ (*i*) with *X*(*i*) as follows. If *X*^*new*^ (*i*) is better than *X*(*i*), then *X*(*i*) will be replaced by *X*^*new*^ (*i*). Thus, the population *X* will be updated. It is worth noting that the evaluations only need be operated in this step.
**Step 6**: Find the best individual of *X*, i.e., *x*_*best*_.
**Step 7**: Use the maximum number of function evaluations (*MaxFEs*) as the terminal condition. If the number of function evaluations (FEs) is larger than *MaxFEs*, then the algorithm terminates. Otherwise, go to **Step 2** for another round of iteration.

### 3.1 Sub-population division method

In PRO, the whole population is divided into the poor and the rich sub-populations, where the former comprises the half of those individuals having the worse fitness values in the entire population. The poor has a slower convergence speed than the rich, but they can help the latter explore more new solutions and quickly move to the optimum solution, by providing abundant evolutionary information. In sum, the poor sub-population focuses on maintaining population diversity, whereas the rich sub-population is mainly responsible for search and exploration.

Generally, each evolutionary stages has different requirements for algorithm performance. At the initial stage, evolution algorithms usually are expected to quickly move to the region belonging to the optimal solution, given the favorable swarm diversity. Along with the evolution processing, the difference in the fitness values between individuals continuously decreases, that is, these individuals become increasingly similar, thereby, gradually reducing population diversity. In this case, the IPRO algorithm can increase population diversity to escape the local optimum. To allow this algorithm, to satisfy the requirements of different evolutionary stages, at the initial stage of evolution, the size of the poor sub-population should be appropriately reduced and that of the rich sub-population should be expanded. Meanwhile, at the final stage of evolution, the size of the poor sub-population should be expanded, whereas that of the poor sub-population should be reduced.

From the aforementioned ideas, this section proposes the following dynamic division method. In each iteration, the individuals of the offspring population are sorted in a descending order based on their fitness values for the minimum optimization problems. Those individuals with z smaller fitness values moved to the rich sub-population, where z is calculated by Eq (5), whereas the other individuals (*N-Z)* moved to the rich sub-population.

$$z=\left\lfloor z_{\text{max}}-(z_{\text{max}}-z_{\text{min}})\times ((t-1)/(T-1))\right\rfloor$$ (5)

where ⌊ ⌋ represents round off, *Z* represents the size of the rich sub-population (the size of the poor sub-population should be *N-Z*), *t* and *T* represent the current and maximum number of iterations, *z*_*max*_ and *z*_*min*_ represent the minimum and maximum sizes of the rich sub-population, respectively. When *z*_*max*_ and *z*_*min*_ are set to *0*.*6N* and *0*.*4N*, respectively, PRO can obtain satisfactory results.

### 3.2 Improved individual updating mechanism for the rich

Eq (2) shows that each rich individual in the PRO algorithm is improved in the following way. The rich sub-population increases its difference from the poor sub-population by learning from the individual in the poor population with the best fitness values. Unfortunately, experiments suggest that the above individual updating mechanism for the rich has a slow convergence speed and can easily fall in the local optimum for two reasons. First, all individuals in the rich sub-population learn from the same individual in the poor sub-population. Despite being the best in its sub-population, the evolutionary information of this individual is not the best in the current population. Obviously, those individuals in the rich sub-population have low probability of searching for better solutions, which would slow down the convergence speed to some extent. Second, given that all individuals in the rich sub-population learn from the same individual in the poor sub-population, they become increasingly similar to each other, which in turn increases the possibility of the PRO algorithm to fall in the local optimum. Moreover, if the optimal individual in the poor sub-population remains unchanged after many iterations, the rich sub-population will converge extremely slowly. The PRO algorithm cannot easily escape the local optimum in this case.

Given that the excellent individuals in the rich sub-population carry the best evolutionary information of the current population, the other members of the same sub-population can converge quickly by learning from these excellent individuals. To further accelerate the convergence speed and maintain population diversity, the rich should learn from both the poor and the better individuals in its own sub-population. Given that the PRO algorithm has different requirements for convergence speed and population diversity at different stages of iterations, at the initial stage of evolution, the individuals in the rich sub-population should learn more from the excellent individuals in the same sub-population rather than from the poor sub-population, whereas at the final stage of evolution, these individuals should learn more from the poor sub-population. Following the above ideas, this study designs the following individual updating mechanisms for the rich sub-population as Eq (6).

$$\begin{array}{ll} X\_rich^{new}(i)= & X\_rich^{old}(i)+r1\times (X\_rich^{old}(k1)-X\_rich^{old}(i))\\ & \text{+}\omega^{t}\times r2\times (X\_rich^{old}(i)-X\_poor^{old}(k2)) \end{array}$$ (6)

where *X*_*rich*^*old*^(*k*1) represents an individual that is randomly selected from the *s* best individuals in the rich sub-population, generally (a good result can be obtained when *s* equals 10), *X*_*poor*^*old*^(*k*2) represents an individual that is randomly selected from the poor sub-population, *r1* and *r2* represent a matrix with one row and *D* columns that composes a random number between 0 and 1, and the parameter *ω*^*t*^ controls the learning proportion, and changes adaptively along with the iteration according to Eq (7).

$$\omega^{t}=\omega_{l}+(1-\frac{t-1}{T-1})\times (\omega_{u}-\omega_{l})$$ (7)

where *ω*_*u*_ and *ω*_*l*_ are the upper and lower terms of *ω*^*t*^, respectively. A good result can be generally obtained, when *ω*_*u*_ and *ω*_*l*_ are set to 1 and 0.2, respectively.

In sum, this section proposes a novel individuals updating mechanism for the rich sub-population. This mechanism uses the original rich and poor sub-populations as its inputs and generates a new rich sub-population as its output. Each individual in the new rich sub-population is generated as follows. First, an individual is randomly selected from the *s* best individual in the original rich sub-population. Second, an individual is randomly selected from the original poor sub-population. Third, *ω*^*t*^ is calculated using Eq (7). Fourth, an individual in the new rich sub-population is generated using Eq (5). It is worth noting that, all individuals in the new rich sub-population need not be evaluated in.this step.

### 3.3 Improved individual updating mechanism for the poor

As shown in Eq (3), each individual in the poor sub-population learn from the same individual that is calculated by the rich in every generation. As the iterations progress, the genes of individuals in the poor sub-population become increasingly similar to those of individuals in the rich sub-population, thereby reducing population diversity. To escape the local optimum, the poor offers less help to the rich, as they could no longer easily provide other evolutionary information. To reduce the possibility of falling into the local optimum and maintain a fast convergence speed, abundant and excellent evolutionary information must be provided for the rich.

From the above ideas, an improved individual updating mechanism for the poor is proposed as follow. First, the individuals in the poor sub-population perform a Gaussian mutation operation on themselves using Eq (8). Second, after each p iterations, the individuals in the poor sub-population learn from the rich once according to Eq (10). Third, a new individual is generated according to Eq (11).

$$X\_poor^{new}(i)=X\_poor^{old}(i)\times (1+q\times randn(1,D))$$ (8)

where the parameter *q* is calculated as

$$q=\{\begin{array}{ll} 1, & if\;f(X\_poor^{old}(i))<f(X\_poor^{old}(k4))\\ {\text{e}}^{\frac{f(X\_poor^{old}(k4))-f(X\_poor^{old}(i))}{\left|f(X\_poor^{old}(i))+0.0001\right|}}, & else \end{array}$$ (9)

where *f*(*X*_*poor*^*old*^(*i*)) and *f*(*X*_*poor*^*old*^(*k*4)) represent the fitness values of the current individual and the other individuals selected randomly in the poor sub-population, respectively.

$$X\_poor^{new}(i)=X\_poor^{old}(i)+r\times (X\_rich^{old}(k3)-X\_poor^{old}(i))$$ (10)

where *X*_*rich*^*old*^(*k*3) is an individual that is selected randomly from the original rich sub-population, and r is a random number between *R1* and *R2*. A good result is generally obtained, when *R1* and *R2* are set to 0.2 and 0.8, respectively.

$$X\_poor^{new}(i,j)=\{\begin{array}{ll} X\_poor^{new}(i,j), & if\;rand<cr\\ X\_poor^{old}(i,j), & else \end{array}$$ (11)

where *X*_*poor*^*new*^ (*i*) and *X*_*poor*^*old*^ (*i*) represent the new individual generated by Eqs (8) or (10) and the original individual, respectively, *j* represents the *j*-th dimension, and *cr* is a crossover probability. A good result is generally obtained, when *cr* is set to 0.4.

Algorithm 3 give the pseudo code of the proposed improved individuals updating mechanism for the poor sub-population. It is worth noting that, all new individuals need not be evaluated in this mechanism.

**Algorithm 3**: the novel improved individuals updating mechanism for the poor sub-population

**Input**: *X*_*rich*^*old*^, *X*_*poor*^*old*^, *p*, *t // X*_*rich*^*old*^ and *X*_*poor*^*old*^ represent the orignal rich sub-population, the original poor sub-population, respectively.

**Output:** *X*_*poor*^*new*^// the new poor sub-population

01 for i = 1 to *N*-Z

02  if mod(t,p) = = 0

03   *k*3 ← select an individual randomly d from the original rich sub-population

04   *X*_*poor*^*new*^ (*i*) ← generate a new individual according to Eq (10)

05  **else**

06   *q* ← obtain according to Eq (9)

07   *X*_*poor*^*new*^(*i*) ← generate a new individual according to Eq (8)

08  end if

09 *X*_*poor*^*new*^ (*i*) ← perform Eq (11) on *X*_*poor*^*new*^ (*i*) and *X*_*poor*^*old*^ (*i*)

10 end for

11 return *X*_*poor*^*new*^

In sum, our proposed improved individual updating method for the poor sub-population has the following two advantages. On the one hand, the individuals in the poor sub-population learn from the rich every *p* iterations rather than each iterations, which not only improves the evolutionary information of the poor sub-population to some extent, but also minimizes losses in their own population diversity. Therefore, the poor can provide more excellent evolutionary information to the rich, helping them quickly move to the region of the optimal solution. On the other hand, the poor performs a Gaussian mutation operation in most iterations, which makes them perform a search around themselves. In addition, most of the original evolution information can be retained according to the crossover mechanism shown in Eq (11). These operations further maintain the population diversity. Therefore, despite falling into the local optimum, the PRO algorithm also has an increased possibility of jumping out.

## 4. Experimental results and discussions

In this section, the following four experiments were performed to investigate the performance of our proposed IPRO algorithm: (1) sensitivity analysis with the IPRO parameters; (2) Verification the effectiveness of each improved strategy proposed in Sections 3.1 to 3.3; (3) the optimization performance comparison between IPRO and original PRO; (4) performance comparison between IPRO and other superior swarm intelligence algorithms. The above experiments were performed on the CEC2013 test suite, which contains 28 functions. According to their characteristics, these functions can be divided into unimodal (F1—F5), multimodal (F6—F20), and composition functions (F21—F28). Details of the test suite can be found in [32]. The above experiments were carried out on MATLAB 2016.

### 4.1 Sensitivity analysis with the IPRO parameters

Compared with the original PRO algorithm, our proposed IPRO algorithm adds the parameters *z*_*min*_, *z*_*max*_, *ω*_*l*_, *ω*_*u*_, *R1*, *R2*, *cr*, *s*, and *p*. A sensitivity analysis was performed to evaluate the influence of these parameters on the performance of the IPRO algorithm. When analyzing the sensitivity of each parameter, only the value of one parameter was changed to ensure that the other parameters remain unchanged. In each experiment, the population size *N* was set to 100, the problem dimension *D* was set to 30, the maximum evaluation times MaxFEs was set to 10000×*D =* 300000, and *Pmut* was set to 0.06 similar to that in the original PRO algorithm.

1. In the sensitivity analysis with *z*_*min*_ and *z*_*max*_, the parameters were set as follows. *z*_*min*_ and *z*_*max*_ correspond to three groups of values, namely, *z*_*min*_ = 0.4 and *z*_*max*_ = 0.6, *z*_*min*_ = 0.3 and *z*_*max*_ = 0.7, *z*_*min*_ = 0.2 and *z*_*max*_ = 0.8. The other parameters in IPRO, except for *z*_*min*_ and *z*_*max*_, were set as *ω*_*l*_ = 0.2, *ω*_*u*_ = 1, *R1 =* 0.2, *R2 =* 0.8, *cr* = 0.4, *s* = 10, and *p* = 10.
2. In the sensitivity analysis with *ω*_*l*_ and *ω*_*u*_, the parameters were set as follows. *ω*_*l*_ and *ω*_*u*_ correspond to three groups of values, namely, *ω*_*l*_ = 0.2 and *ω*_*u*_ = 1, *ω*_*l*_ = 0.2 and *ω*_*u*_ = 0.8, *ω*_*l*_ = 0.4 and *ω*_*u*_ = 0.6. The other parameters in IPRO, except for *ω*_*l*_ and *ω*_*u*_, are set as follows: *z*_*min*_ = 0.4, *z*_*max*_ = 0.6, *R1 =* 0.2, *R2 =* 0.8, *cr* = 0.4, *s* = 10, and *p* = 10.
3. In the sensitivity analysis with *cr*, the values of *cr* were set to 0.2,0.4,0.6, respectively, and the other parameters were set as *ω*_*l*_ = 0.2, *ω*_*u*_ = 1, *z*_*min*_ = 0.4, *z*_*max*_ = 0.6, *R1 =* 0.2, *R2 =* 0.8, *s* = 10, and *p* = 10.
4. In the sensitivity analysis with *R1* and *R2*, the parameters were set as follows. *R1* and *R2* correspond to three groups of values, namely, R1 = 0.2 and R2 = 1, R1 = 0.2 and R2 = 0.8, R1 = 0.4 and R2 = 0.6. The other parameters in IPRO, except for *R1* and *R2*, were set as follows: *ω*_*l*_ = 0.2, *ω*_*u*_ = 1, *z*_*min*_ = 0.4, *z*_*max*_ = 0.6, *R1 =* 0.2, *R2 =* 0.8, *cr* = 0.4, *s* = 10, and *p* = 10.
5. In the sensitivity analysis with *s*, the values of *s* were set to 10, 50, 100, respectively, and the other parameters were set as follows: *ω*_*l*_ = 0.2, *ω*_*u*_ = 1, *z*_*min*_ = 0.4, *z*_*max*_ = 0.6, *R1 =* 0.2, *R2 =* 0.8, *cr* = 0.4, and *p* = 10.
6. In the sensitivity analysis with *p*, the values of *p* were set to 10, 50, 100, respectively, and the other parameters were set as follows: *ω*_*l*_ = 0.2, *ω*_*u*_ = 1, *z*_*min*_ = 0.4, *z*_*max*_ = 0.6, *R1 =* 0.2, *R2 =* 0.8, *cr* = 0.4, and *s* = 10.

The IPRO algorithm with the above parameter settings ran independently 30 times on the CEC2013 test suite. Tables 1 and 2 present the average optimal values obtained in each run with the above different parameter settings when the same preset MaxFEs is reached. The last line of these tables presents the number of functions after the algorithm with the corresponding parameter settings achieves the best optimization effect. The IPRO with *z*_*min*_ = 0.2 and *z*_*max*_ = 0.8 obtained 14 out of 28 functions optimum, the IPRO with *z*_*min*_ = 0.3 and *z*_*max*_ = 0.7 obtained 12 out of 28 functions optimum, and the IPRO with *z*_*min*_ = 0.4 and *z*_*max*_ = 0.6 obtained 22 out of 28 functions optimum. In sum, the performance of IPRO is sensitive to *z*_*min*_ and *z*_*max*_. Meanwhile, the other data in Tables 1 and 2 show that the performance of IPRO is sensitive to *ω*_*l*_, *ω*_*u*_, cr, and s, and is less sensitive to *R1*, *R2*, and *p*.

**Table 1:** Sensitivity analysis with *z*_min_, *z*_*max*_, *ω*_*l*_, *ω*_*u*_, *cr*.

	*z*_min_, *z*_max_			*ω*_*l*_, *ω*_*u*_			*cr*		
	0.2,0.8	0.3,0.7	0.4,0.6	0.2,0.8	0.2,1	0.4,1	0.1	0.4	0.7
F1	1.68E-30	3.79E-30	**0.00E+00**	**0.00E+00**	**0.00E+00**	**0.00E+00**	1.68E-30	**0.00E+00**	**0.00E+00**
F2	4.00E+06	3.77E+06	**5.78E+05**	3.34E+06	**5.78E+05**	3.17E+06	3.36E+06	**5.78E+05**	3.43E+06
F3	**0.00E+00**	**0.00E+00**	**0.00E+00**	**0.00E+00**	**0.00E+00**	**0.00E+00**	**0.00E+00**	**0.00E+00**	**0.00E+00**
F4	6.86E+03	6.80E+03	**2.87E+03**	5.95E+03	**2.87E+03**	9.99E+03	7.37E+03	**2.87E+03**	7.70E+03
F5	**0.00E+00**	**0.00E+00**	**0.00E+00**	**0.00E+00**	**0.00E+00**	**0.00E+00**	**0.00E+00**	**0.00E+00**	**0.00E+00**
F6	1.94E+01	1.73E+01	**1.20E+01**	1.71E+01	**1.20E+01**	1.85E+01	1.75E+01	**1.20E+01**	1.83E+01
F7	**0.00E+00**	**0.00E+00**	**0.00E+00**	**0.00E+00**	**0.00E+00**	**0.00E+00**	**0.00E+00**	**0.00E+00**	**0.00E+00**
F8	2.10E+01	2.10E+01	**2.09E+01**	**2.09E+01**	**2.09E+01**	**2.09E+01**	2.10E+01	**2.09E+01**	**2.09E+01**
F9	**0.00E+00**	**0.00E+00**	**0.00E+00**	**0.00E+00**	**0.00E+00**	**0.00E+00**	**0.00E+00**	**0.00E+00**	**0.00E+00**
F10	6.00E-02	**1.17E-02**	3.01E-02	**1.23E-02**	3.01E-02	1.36E-02	1.46E-02	3.01E-02	**8.00E-03**
F11	**0.00E+00**	**0.00E+00**	**0.00E+00**	**0.00E+00**	**0.00E+00**	**0.00E+00**	**0.00E+00**	**0.00E+00**	**0.00E+00**
F12	**0.00E+00**	**0.00E+00**	**0.00E+00**	**0.00E+00**	**0.00E+00**	**0.00E+00**	**0.00E+00**	**0.00E+00**	**0.00E+00**
F13	**0.00E+00**	**0.00E+00**	**0.00E+00**	**0.00E+00**	**0.00E+00**	**0.00E+00**	**0.00E+00**	**0.00E+00**	**0.00E+00**
F14	**6.21E+03**	6.24e+03	6.51E+03	6.63E+03	**6.51E+03**	6.63E+03	**6.33E+03**	6.51E+03	6.53E+03
F15	7.21E +03	7.08E+03	**6.97E+03**	7.00E+03	**6.97E+03**	7.08E+03	7.10E+03	**6.97E+03**	7.07E+03
F16	**2.42E+00**	2.46E+00	2.47E+00	2.53E+00	**2.47E+00**	2.50E+00	2.51E+01	**2.47E+00**	2.52E+01
F17	1.69E+02	1.71E+02	**1.48E+02**	1.61E+02	**1.48E+02**	1.70E+02	1.63E+02	**1.48E+02**	1.70E+02
F18	1.88E+02	1.89E+02	**1.84E+02**	**1.82E+02**	1.84E+02	1.89E+02	**1.84E+02**	**1.84E+02**	1.92E+02
F19	1.37E+01	1.39E+01	**9.44E+00**	1.31E+01	**9.44E+00**	1.39E+01	1.31E+01	**9.44E+00**	1.33E+01
F20	**0.00E+00**	**0.00E+00**	**0.00E+00**	**0.00E+00**	**0.00E+00**	**0.00E+00**	**0.00E+00**	**0.00E+00**	**0.00E+00**
F21	**3.73E+02**	3.87E+02	4.00E+02	**4.00E+02**	**4.00E+02**	**4.00E+02**	**3.93E+02**	4.00E+02	4.00E+02
F22	6.02E+03	**5.83E+03**	6.05E+03	6.16E+03	6.05E+03	**5.91E+03**	**5.87E+03**	6.05E+03	6.07E+03
F23	7.06E+03	7.10E+03	**6.92E+03**	**6.90E+03**	6.92E+03	6.99E+03	**6.91E+03**	6.92E+03	6.93E+03
F24	**2.00E+02**	**2.00E+02**	**2.00E+02**	**2.00E+02**	**2.00E+02**	**2.00E+02**	**2.00E+02**	**2.00E+02**	**2.00E+02**
F25	2.44E+02	2.50E+02	**2.42E+02**	2.53E+02	**2.42E+02**	2.48E+02	2.74E+02	2.42E+02	**2.40E+02**
F26	2.77E+02	2.81E+02	**2.58E+02**	2.79E+02	**2.58E+02**	2.79E+02	**2.51E+02**	2.58E+02	2.66E+02
F27	**1.61E+03**	1.63E+03	**1.61E+03**	1.64E+03	**1.61E+03**	1.63E+03	1.62E+03	**1.61E+03**	**1.61E+03**
F28	**8.16E+02**	**8.16E+02**	8.25E+02	**8.14E+02**	8.25E+02	8.19E+02	8.19E+02	8.25E+02	**8.18E+02**
	14	12	**22**	16	**23**	13	15	**20**	15

**Table 2:** Sensitivity analysis with *R1*, *R2*, *s*, *p*.

	*R1*, *R2*			s			*p*		
	0.2,0.8	0.2,1	0.4,0.8	5	10	20	5	10	20
F1	**0.00E+00**	**0.00E+00**	**0.00E+00**	**0.00E+00**	**0.00E+00**	4.30E-26	**0.00E+00**	**0.00E+00**	**0.00E+00**
F2	5.78E+05	1.01E+06	**1.86E+05**	1.74E+06	**5.78E+05**	8.90E+06	6.06E+05	**5.78E+05**	1.35E+06
F3	**0.00E+00**	**0.00E+00**	**0.00E+00**	**0.00E+00**	**0.00E+00**	**0.00E+00**	**0.00E+00**	**0.00E+00**	**0.00E+00**
F4	**2.87E+03**	4.42E+03	3.02E+03	4.05E+03	**2.87E+03**	1.09E+04	**2.07E+03**	2.87E+03	6.14E+03
F5	**0.00E+00**	**0.00E+00**	**0.00E+00**	**0.00E+00**	**0.00E+00**	8.30E-19	**0.00E+00**	**0.00E+00**	1.44E-24
F6	1.20E+01	1.59E+01	**8.48E+00**	2.22E+01	**1.20E+01**	1.71E+01	**1.11E+01**	1.20E+01	1.59E+01
F7	**0.00E+00**	**0.00E+00**	**0.00E+00**	**0.00E+00**	**0.00E+00**	**0.00E+00**	**0.00E+00**	**0.00E+00**	**0.00E+00**
F8	**2.09E+01**	2.10E+01	2.10E+01	2.10E+01	**2.09E+01**	**2.09E+01**	2.10E+01	**2.09E+01**	2.10E+01
F9	**0.00E+00**	**0.00E+00**	**0.00E+00**	**0.00E+00**	**0.00E+00**	**0.00E+00**	**0.00E+00**	**0.00E+00**	**0.00E+00**
F10	3.01E-02	**1.40E-02**	2.03E-02	2.33E-02	3.01E-02	**4.90E-03**	2.64E-02	3.01E-02	**7.10E-03**
F11	**0.00E+00**	**0.00E+00**	**0.00E+00**	**0.00E+00**	**0.00E+00**	**0.00E+00**	**0.00E+00**	**0.00E+00**	**0.00E+00**
F12	**0.00E+00**	**0.00E+00**	**0.00E+00**	**0.00E+00**	**0.00E+00**	**0.00E+00**	**0.00E+00**	**0.00E+00**	**0.00E+00**
F13	**0.00E+00**	**0.00E+00**	**0.00E+00**	**0.00E+00**	**0.00E+00**	**0.00E+00**	**0.00E+00**	**0.00E+00**	**0.00E+00**
F14	6.51E+03	6.42E+03	**6.40E+03**	6.46E+03	6.51E+03	**6.42E+03**	**6.42E+03**	6.51E+03	**6.28E+03**
F15	**6.97E+03**	7.15E+03	7.10E+03	7.11E+03	**6.97E+03**	7.17E+03	7.06E+03	**6.97E+03**	7.14E+03
F16	**2.47E+00**	2.52E+00	2.59E+00	**2.44E+00**	2.47E+00	2.46E+00	2.58E+00	**2.47E+00**	2.51E+00
F17	**1.48E+02**	1.54E+02	1.60E+02	**1.37E+02**	1.48E+02	1.78E+02	1.52E+02	**1.48E+02**	1.75E+02
F18	**1.84E+02**	1.86E+02	**1.84E+02**	1.86E+02	**1.84E+02**	1.94E+02	1.84E+02	1.84E+02	**1.82E+02**
F19	9.44E+00	1.23E+01	**7.60E+00**	1.05E+01	**9.44E+00**	1.41E+00	**7.20E+00**	9.44E+00	1.36E+01
F20	**0.00E+00**	**0.00E+00**	**0.00E+00**	**0.00E+00**	**0.00E+00**	**0.00E+00**	**0.00E+00**	**0.00E+00**	**0.00E+00**
F21	**4.00E+02**	**4.00E+02**	**4.00E+02**	4.00E+02	4.00E+02	**3.93E+02**	**4.00E+02**	**4.00E+02**	**4.00E+0**2
F22	6.05E+03	**6.00E+03**	**6.00E+03**	**5.93E+03**	6.05E+03	6.20E+03	6.11E+03	**6.05E+03**	**6.05E+03**
F23	**6.92E+03**	6.97E+03	6.97E+03	**6.80E+03**	6.92E+03	6.94E+03	**6.86E+03**	6.92E+03	7.07E+03
F24	**2.00E+02**	**2.00E+02**	**2.00E+02**	**2.00E+02**	**2.00E+02**	**2.00E+02**	**2.00E+02**	**2.00E+02**	2.00E+02
F25	2.42E+02	**2.41E+02**	2.42E+02	2.49E+02	**2.42E+02**	2.47E+02	**2.42E+02**	**2.42E+02**	2.51E+02
F26	**2.58E+02**	2.92E+02	2.70E+02	2.75E+02	**2.58E+02**	2.75E+02	2.86E+02	**2.58E+02**	2.75E+02
F27	**1.61E+03**	**1.61E+03**	**1.61E+03**	1.64E+03	**1.61E+03**	**1.61E+03**	**1.61E+03**	**1.61E+03**	**1.61E+03**
F28	8.25E+02	8.23E+02	**8.21E+02**	8.33E+02	8.25E+02	**8.16E+02**	**8.18E+02**	8.25E+02	8.19E+02
	20	15	19	14	**20**	**14**	19	**20**	**14**

### 4.2 Verification of the effectiveness of various improvement measures

To verify the effectiveness of each improvement measure described in Sections 3.1 to 3.3, the following experiments were conducted. First, the PRO algorithm was combined with each of the improved strategies proposed in Sections 3.1 to 3.2, which yielded three new algorithms, namely, an IPRO algorithm based on the dynamic population division method proposed in Section 3.1(PRO1), an IPRO algorithm based on the individual updating mechanism for the rich proposed in Section 3.2(PRO2), and an IPRO algorithm based on the individual updating mechanism for the poor proposed in Section 3.3 (PRO3). These new algorithms were then tested on the CEC2013 test suite. To ensure the fairness of comparison, in each experiment, the population size *N* were set to 100, the problem dimension *D* were set to 30, and the maximum number of evaluations *MaxFes* = 10000 × *D* = 300000, the other parameters were set as follows: *Pmut* = 0.06, *z*_min_ = 0.4, *z*_max_ = 0.6, *ω*_*l*_ = 0.2, *ω*_*u*_ = 1, *R1 =* 0.2, *R2 =* 0.8, *cr* = 0.4, *s* = 10, and *p* = 10. To avoid the contingency of a single operation of the algorithm and adversely affect the evaluation of the algorithm. Each algorithm runs independently on each function 30 times.

Table 3 presents the mean and standard deviation of the optimal values obtained by PRO1, PRO2, PRO3, and PRO on each function in 30 experiments. The data before and after the symbol “±” represents mean and standard deviation respectively. To prove that the results achieved by the above algorithms were not obtained by chance, the results shown in Table 3 were subjected to a Wilcoxon rank sum test. The test results were then used to determine whether significant differences were present (alternative hypothesis) or absent (null hypothesis) among these algorithms at the 0.05 significance level on each optimization function. The Wilcoxon rank sum test was used in this work because of its fewer distributional assumptions compared with other parametric procedures, such as the T-test. Table 4 presents the Wilcoxon rank sum test results. A p-value of less than 0.05 indicates the presence of a significant difference between the IPRO and PRO on the current function; otherwise, it indicates there is no significant difference between IPRO and PRO in the current function. The symbols ‘+, =, and -’ in the last line of Table 4 indicate the number of functions on which the IPRO significantly outperforms the PRO, on which the IPRO has no significant differences from the PRO, and on which the PRO significantly outperforms the IPRO.

**Table 3:** Comparison results of each improved strategy on the 30-dimensional CEC2013 test suite.

	PRO	PRO1	PRO2	PRO3
F1	9.06E+00±2.10E+00	9.80E+00±3.07E+00	**2.20E-04**±**1.25E-04**	1.09E+01±2.68E+00
F2	4.35E+06±2.40E+06	**3.87E+06**±**1.65E+06**	**4.29E+05**±**1.46E+05**	1.71E+07±2.80E+06
F3	**0.00E+00**±**0.00E+00**	**0.00E+00**±**0.00E+00**	**0.00E+00**±**0.00E+00**	**0.00E+00**±**0.00E+00**
F4	1.01E+04±3.73E+03	1.07E+04±4.44E+03	**1.44E+03**±**5.21E+02**	4.14E+04±4.47E+03
F5	2.33E+01±1.25E+01	2.67E+01±1.41E+01	**1.14E-03**±**4.10E-04**	3.01E+02±8.30E+01
F6	7.90E+01±2.48E+01	**6.67E+01**±**3.08E+01**	**1.00E+01**±**8.99E+00**	1.12E+02±1.51E+01
F7	9.78E+00±2.57E+01	**9.01E+00**±**2.56E+01**	**0.00E+00**±**0.00E+00**	**0.00E+00**±**0.00E+00**
F8	2.10E+01±3.68E-02	**2.10E+01**±**4.32E-02**	**2.09E+01**±**5.48E-02**	**2.10E+01**±**3.88E-02**
F9	2.82E+01±1.17E+01	2.98E+01±1.03E+01	**7.45E+00**±**1.25E+01**	**0.00E+00**±**0.00E+00**
F10	6.45E+00±1.81E+00	7.19E+00±1.78E+00	**2.83E-02**±**1.65E-02**	5.62E+01±1.32E+01
F11	8.91E+01±5.28E+01	1.07E+02±6.50E+01	**3.52E+00**±**1.04E+01**	**5.69E-01**±**1.29E+00**
F12	2.46E+02±5.82E+01	2.84E+02±7.53E+01	**1.86E+01**±**4.76E+01**	**0.00E+00**±**0.00E+00**
F13	2.74E+02±5.43E+01	**2.65E+02**±**9.52E+01**	**1.44E+01**±**4.34E+01**	**4.09E-01**±**2.20E+00**
F14	7.54E+03±3.23E+02	**6.95E+03**±**1.11E+03**	**6.50E+03**±**4.22E+02**	**6.58E+03**±**3.54E+02**
F15	6.22E+03±1.25E+03	**5.65E+03**±**1.15E+03**	**5.07E+03**±**1.44E+03**	6.65E+03±2.09E+02
F16	2.32E+00±2.84E-01	2.36E+00±2.61E-01	2.45E+00±2.88E-01	**2.22E+00**±**2.55E-01**
F17	6.53E+02±1.83E+02	6.87E+02±1.61E+02	**1.89E+02**±**4.85E+01**	**2.87E+02**±**3.98E+01**
F18	6.94E+02±1.76E+02	**6.85E+02**±**1.46E+02**	**2.04E+02**±**1.92E+01**	**3.02E+02**±**3.06E+01**
F19	2.81E+01±5.06E+00	**2.79E+01**±**4.84E+00**	**1.37E+01**±**1.40E+00**	**2.08E+01**±**3.44E+00**
F20	1.48E+01±4.82E-01	**1.30E+01**±**1.43E+00**	**2.83E-01**±**1.53E+00**	**3.21E+00**±**3.88E+00**
F21	4.02E+02±3.73E-01	4.02E+02±4.69E-01	**4.00E+02**±**7.38E-04**	4.02E+02±4.91E-01
F22	8.28E+03±4.24E+02	**8.23E+03**±**4.32E+02**	**6.32E+03**±**4.45E+02**	**7.31E+03**±**2.98E+02**
F23	7.88E+03±6.68E+02	**7.83E+03**±**6.07E+02**	**6.75E+03**±**5.25E+02**	**7.21E+03**±**2.98E+02**
F24	2.47E+02±4.03E+01	2.59E+02±3.76E+01	**2.04E+02**±**1.17E+01**	**2.01E+02**±**8.29E-02**
F25	3.38E+02±1.93E+01	**3.31E+02**±**1.46E+01**	**2.56E+02**±**3.35E+01**	**2.36E+02**±**1.10E+01**
F26	3.64E+02±2.50E+01	**3.49E+02**±**4.43E+01**	**3.03E+02**±**1.93E+01**	**2.59E+02**±**3.41E+01**
F27	2.42E+03±2.98E+02	2.50E+03±1.52E+02	**1.67E+03**±**1.71E+02**	**1.63E+03**±**4.29E+01**
F28	3.19E+03±1.17E+03	3.54E+03±1.05E+03	**8.95E+02**±**3.28E+02**	**1.08E+03**±**5.22E+01**

**Table 4:** Wilcoxon rank sum test between PRO and each improved strategy.

	p-value(vs.PRO)		
	PRO1	PRO2	PRO3
F1	0.271 (=)	0.000(+)	0.005(-)
F2	0.813 (=)	0.000(+)	0.000(-)
F3	1.000 (=)	1.000 (=)	1.000 (=)
F4	0.739 (=)	0.000(+)	0.000(-)
F5	0.274 (=)	0.000(+)	0.000(-)
F6	0.469 (=)	0.000(+)	0.000(-)
F7	0.561 (=)	0.011(+)	0.011(+)
F8	0.441 (=)	0.147 (=)	0.795 (=)
F9	0.529 (=)	0.000(+)	0.000(+)
F10	0.130 (=)	0.000(+)	0.000(+)
F11	0.333 (=)	0.000(+)	0.000(+)
F12	0.051 (=)	0.000(+)	0.000(+)
F13	0.072 (=)	0.000(+)	0.000(+)
F14	0.045(+)	0.000(+)	0.000(+)
F15	0.076 (=)	0.002(+)	0.796 (=)
F16	0.544 (=)	0.121 (=)	0.169 (=)
F17	0.379 (=)	0.000(+)	0.000(+)
F18	0.865 (=)	0.000(+)	0.000(+)
F19	0.918 (=)	0.000(+)	0.000(+)
F20	0.000(+)	0.000(+)	0.000(+)
F21	0.801 (=)	0.000(+)	0.002(-)
F22	0.464 (=)	0.000(+)	0.000(+)
F23	0.631 (=)	0.000(+)	0.000(+)
F24	0.254 (=)	0.000(+)	0.000(+)
F25	0.052 (=)	0.000(+)	0.000(+)
F26	0.270 (=)	0.000(+)	0.000(+)
F27	0.264 (=)	0.000(+)	0.000(+)
F28	0.128 (=)	0.000(+)	0.000(+)
+/ = /-	2/26/0	25/3/0	18/4/6

Compared with PRO, PRO1 demonstrated the same performance on 26 functions, a significantly better performance on the multimodal function F14 and composition function F20, and a significantly inferior performance on any function. Meanwhile, PRO2 obtains smaller mean values on 27 functions, the same performance on F3, F8 and F16, a significantly better performance on 25 functions, a significantly inferior performance on 6 functions, and a significantly better performance on 18 test functions.

In sum, the strategies proposed in Sections 3.1 to 3.3, greatly influenced the improvements in algorithm accuracy with the strategy proposed in Section 3.2 showing the most obvious improvement effect.

### 4.3 Performance comparison between the IPRO and PRO

To compare the performance of our proposed IPRO and the original PRO, they were tested on 10- and 30-dimensional CEC2013 test suites. To ensure the fairness of comparison, the population size *N* was set to 100, and the maximum number of evaluations was set to *MaxFes =* 300000. The other parameters were set following those described in section 4.1. Table 5 presents the results of 30 independent experiments conducted on the 10- and 30-dimensional CEC2013 test suites, where “mean” and “std” indicate the mean and standard deviation, and “p-value (IPRO vs. PRO)” in the fourth and seventh columns indicate the Wilcoxon rank sum test results of the algorithms on the 10- and 30-dimensional functions, respectively.

**Table 5:** Comparison results of IPRO and PRO on CEC2013 test suite.

function	D = 10					D = 30				
	PRO		IPRO		*p-value*	PRO		IPRO		*p-value*
	mean	std	mean	std	(IPROvs.PRO)	mean	std	mean	std	(IPROvs.PRO)
F1	1.07E-03	9.86E-04	**0.00E+00**	**0.00E+00**	0.000 (+)	9.06E+00	2.10E+00	**0.00E+00**	**0.00E+00**	0.000 (+)
F2	**8.31E+03**	**1.75E+04**	1.20E+04	1.29E+04	0.014 (-)	4.35E+06	2.40E+06	**5.78E+05**	**4.20E+05**	0.000 (+)
F3	**0.00E+00**	**0.00E+00**	**0.00E+00**	**0.00E+00**	1.000 (=)	**0.00E+00**	**0.00E+00**	**0.00E+00**	**0.00E+00**	1.000 (=)
F4	**1.88E+01**	**3.33E+01**	3.66E+02	1.79E+02	0.000 (-)	1.01E+04	3.73E+03	**2.87E+03**	**8.71E+02**	0.000 (+)
F5	2.14E-03	1.17E-03	**0.00E+00**	**0.00E+00**	0.000 (+)	2.33E+01	1.25E+01	**0.00E+00**	**0.00E+00**	0.000 (+)
F6	**5.27E+00**	**4.91E+00**	7.86E+00	3.91E+00	0.596 (=)	7.90E+01	2.48E+01	**1.20E+01**	**1.20E+01**	0.000 (+)
F7	**0.00E+00**	**0.00E+00**	**0.00E+00**	**0.00E+00**	1.000 (=)	9.78E+00	2.57E+01	**0.00E+00**	**0.00E+00**	0.011 (+)
F8	5.04E+00	8.41E+00	**4.44E-16**	**2.96E-31**	0.003 (+)	2.10E+01	3.68E-02	**2.09E+01**	**4.62E-02**	0.389 (=)
F9	5.85E-01	1.89E+00	**0.00E+00**	**0.00E+00**	0.082 (=)	2.82E+01	1.17E+01	**0.00E+00**	**0.00E+00**	0.000 (+)
F10	7.36E-01	1.53E-01	**1.18E-02**	**1.97E-02**	0.000 (+)	6.45E+00	1.81E+00	**3.01E-02**	**2.03E-02**	0.000 (+)
F11	3.65E-01	1.61E+00	**0.00E+00**	**0.00E+00**	0.081 (=)	8.91E+01	5.28E+01	**0.00E+00**	**0.00E+00**	0.000 (+)
F12	1.64E+01	2.01E+01	**0.00E+00**	**0.00E+00**	0.000 (+)	2.46E+02	5.82E+01	**0.00E+00**	**0.00E+00**	0.000 (+)
F13	1.16E+01	2.01E+01	**0.00E+00**	**0.00E+00**	0.003 (+)	2.74E+02	5.43E+01	**0.00E+00**	**0.00E+00**	0.000 (+)
F14	1.50E+03	1.70E+02	**8.34E+02**	**9.88E+01**	0.000 (+)	7.54E+03	3.23E+02	**6.51E+03**	**3.65E+02**	0.000 (+)
F15	1.10E+03	2.63E+02	**1.06E+03**	**1.41E+02**	0.108 (=)	**6.22E+03**	**1.25E+03**	6.97E+03	3.32E+02	0.223 (=)
F16	**8.74E-01**	**1.44E-01**	9.46E-01	1.89E-01	0.126 (=)	**2.32E+00**	**2.84E-01**	2.47E+00	3.19E-01	0.039 (-)
F17	7.09E+01	1.87E+01	**2.42E+01**	**2.66E+00**	0.000 (+)	6.53E+02	1.83E+02	**1.48E+02**	**2.39E+01**	0.000 (+)
F18	8.02E+01	2.48E+01	**2.96E+01**	**3.02E+00**	0.000 (+)	6.94E+02	1.76E+02	**1.84E+02**	**1.78E+01**	0.000 (+)
F19	3.81E+00	1.05E+00	**1.37E+00**	**2.23E-01**	0.000 (+)	2.81E+01	5.06E+00	**9.44E+00**	**1.82E+00**	0.000 (+)
F20	3.29E-01	9.13E-01	**0.00E+00**	**0.00E+00**	0.042 (+)	1.48E+01	4.82E-01	**0.00E+00**	**0.00E+00**	0.000 (+)
F21	4.15E+02	3.99E+01	**3.89E+02**	**2.79E+01**	0.003 (+)	4.02E+02	3.73E-01	**4.00E+02**	**0.00E+00**	0.000 (+)
F22	**1.68E+03**	**1.56E+02**	8.65E+02	1.20E+02	0.000 (-)	8.28E+03	4.24E+02	**6.05E+03**	**4.68E+02)**	0.000 (+)
F23	1.60E+03	1.92E+02	**1.09E+03**	**1.70E+02**	0.000 (+)	7.88E+03	6.68E+02	**6.92E+03**	**2.13E+02**	0.000 (+)
F24	1.28E+02	3.44E+01	**1.00E+02**	**1.64E-02**	0.000 (+)	2.47E+02	4.03E+01	**2.00E+02**	**7.65E-03**	0.000 (+)
F25	1.28E+02	2.79E+01	**1.00E+02**	**0.00E+00**	0.000 (+)	3.38E+02	1.93E+01	**2.42E+02**	**3.20E+01**	0.000 (+)
F26	1.18E+02	1.88E+01	**1.00E+02**	**0.00E+00**	0.000 (+)	3.64E+02	2.50E+01	**2.58E+02**	**3.83E+01**	0.000 (+)
F27	1.63E+03	6.14E+01	**1.61E+03**	**2.82E-03**	0.081 (=)	2.42E+03	2.98E+02	**1.61E+03**	**3.89E-01**	0.000 (+)
F28	6.27E+02	2.73E+02	**3.51E+02**	**1.41E+02**	0.000 (+)	3.19E+03	1.17E+03	**8.25E+02**	**1.85E+01**	0.000 (+)

For the 10-dimensional test functions, compared with PRO, our proposed IPRO demonstrated a significantly inferior performance only on 3 functions (F2, F4, and F22), no significant differences on 8 functions (F3, F6, F7, F9, F11, F15, F16, and F27), and a significantly better performance on 17 functions. For the 30-dimensional test functions, our proposed IPRO demonstrated an inferior performance only on F16, a similar on three functions (F3, F8, and F15), and a significantly better performance on the other 24 functions.

In sum, compared with the original PRO, our proposed IPRO shows a significant advantage in convergence accuracy. This advantage become more obvious as the dimension of the optimization problem increases.

### 4.4 Performance comparison between IPRO and other algorithms

To verify its excellence, our proposed IPRO algorithm, was compared with four state-of-the-art algorithms on the CEC2013 test suite, namely, MSMPSO [50], MSCA [49], ADN-RSN-PSO [48], and IATTP [52]. To ensure fairness of comparison, the population size was set to *N* = 100, the problem dimension was set to *D* = 30, and the maximum number of evaluations was set to *MaxFes* = 10000 × *D* = 300000. The values of the other parameters are presented in Table 6.

**Table 6:** Initial parameters setting of each algorithm.

Algorithm	Parameter
MSMPSO	*Pop*_1_ = *Pop*_2_ = *Pop*_3_ = 20, Pop_1_: *c*_1_ = 2.0, *c*_2_ = 1.0, *c*_3_ = 0.2, Pop_2_: *c*_1_ = 0.1, *c*_2_ = 1.0, *c*_3_ = 2.0, Pop_3_: *c*_1_ = 1.0, *c*_2_ = 1.0, *c*_3_ = 1.0, *cycle* = 10
MSCA	*r*_*min*_ = 0.4, *r*_*max*_ = 0.9, *c*_1_ = 0.5, *c*_2_ = 0.5
ADN-RSN-PSO	*χ* = 0.7298, *φ*_1 =_ *φ*_2_ = 2.05, *n*_*s*_ = 2, *s*_*r*_ = 4, *ρ* = 0.4, *P*_*ADN*_ = 0.2, *L*_*o*_ = 0.35 × *range*, *L*_min_ = 10^−8^ × *range*
IATTP	*h*1 = *h*2 = *h*3 = 0.5, *h*4 = 0.8, *m* = 50, *q* = 0.8
IPRO	*Pmut* = 0.06, *z*_min_ = 0.4, *z*_max_ = 0.6, *ω*_*l*_ = 0.2, *ω*_*u*_ = 1, *R*1 = 0.2, *R*2 = 0.8, *cr* = 0.4, *s* = 10, *p* = 10

Table 7 presents the results of 30 independent experiments conducted for each algorithm on the 30-dimensional CEC2013 test suites. To further investigate the improvement gap between IPRO and each state-of-the-art algorithm, these algorithms were subjected to a Wilcoxon rank sum test with a significance level of 0.05, shown in Table 8. The number of functions on which each algorithm significantly outperformed IPRO, on which the IPRO has no significant differences from each algorithm, and on which the PRO significantly outperforms the IPRO, was counted. To a better understanding of the overall performance of these algorithms on scalable test functions, Table 9 shows the Friedman test results for the data presented in Table 7. The algorithm with lower values is given a higher ranking. Further details on Friedman test can be found in [34].

**Table 7:** Comparison results of 5 algorithms on the 30-dimensional CEC2013 test suite.

function	MSMPSO		MSCA		AND-RSN-PSO		IATTP		IPRO	
	mean	standard deviation	mean	standard deviation	mean	standard deviation	mean	standard deviation	mean	standard deviation
F1	2.22E+02	2.08E+02	4.18E+04	4.89E+03	1.34E+02	6.23E+02	8.87E-01	4.08E-01	**0.00E+00**	**0.00E+00**
F2	1.01E+07	5.53E+06	8.74E+08	2.30E+08	8.54E+06	4.71E+06	8.57E+06	3.23E+06	**5.78E+05**	**4.20E+05**
F3	**0.00E+00**	**0.00E+00**	1.11E+18	1.62E+18	**0.00E+00**	**0.00E+00**	**0.00E+00**	**0.00E+00**	**0.00E+00**	**0.00E+00**
F4	2.30E+03	1.07E+03	7.71E+04	6.96E+03	6.05E+04	9.23E+03	**2.64E+02**	**9.94E+01**	2.87E+03	8.71E+02
F5	2.72E+02	9.28E+01	4.52E+04	1.26E+04	6.56E+00	1.52E+01	2.85E+00	1.37E+00	**0.00E+00**	**0.00E+00**
F6	1.42E+02	4.13E+01	8.94E+03	1.54E+03	6.32E+01	3.77E+01	6.99E+01	2.19E+01	**1.20E+01**	**1.20E+01**
F7	9.30E+00	3.15E+01	9.37E+05	1.37E+06	5.61E+04	2.20E+05	**0.00E+00**	**0.00E+00**	**0.00E+00**	**0.00E+00**
F8	2.10E+01	4.78E-02	2.11E+01	6.44E-02	2.14E+01	7.50E-02	2.09E+01	3.84E-02	**2.09E+01**	**4.62E-02**
F9	**0.00E+00**	**0.00E+00**	3.48E+01	2.30E+00	2.57E+01	1.33E+01	**0.00E+00**	**0.00E+00**	**0.00E+00**	**0.00E+00**
F10	6.50E+01	3.41E+01	7.03E+03	1.23E+03	2.29E+02	9.33E+02	1.21E+01	5.17E+00	**3.01E-02**	**2.03E-02**
F11	2.32E-01	7.99E-01	2.42E+02	6.09E+01	**0.00E+00**	**0.00E+00**	**0.00E+00**	**0.00E+00**	**0.00E+00**	**0.00E+00**
F12	1.15E+02	6.49E+01	3.87E+02	4.11E+01	2.47E+02	5.54E+01	**0.00E+00**	**0.00E+00**	**0.00E+00**	**0.00E+00**
F13	8.87E+01	8.33E+01	3.81E+02	4.01E+01	2.74E+02	5.75E+01	**0.00E+00**	**0.00E+00**	**0.00E+00**	**0.00E+00**
F14	**3.86E+03**	**2.68E+02**	8.59E+03	5.83E+02	6.13E+02	2.58E+02	6.54E+03	3.88E+02	6.51E+03	3.65E+02
F15	7.34E+03	3.45E+02	8.66E+03	4.81E+02	5.36E+03	1.56E+03	**6.32E+03**	**6.89E+02**	6.97E+03	3.32E+02)
F16	2.42E+00	2.82E-01	4.16E+00	6.41E-01	2.70E+00	2.00E+00	**2.40E+00**	**3.14E-01**	2.47E+00	3.19E-01
F17	3.83E+02	7.53E+01	1.95E+03	1.54E+02	**2.11E+01**	**1.63E+01**	1.91E+02	1.19E+01	1.48E+02	2.39E+01
F18	3.87E+02	1.07E+02	1.94E+03	1.74E+02	1.12E+03	7.71E+02	2.11E+02	1.96E+01	**1.84E+02**	**1.78E+01**
F19	5.25E+01	2.38E+01	8.28E+05	4.89E+05	1.54E+04	8.29E+04	1.53E+01	2.00E+00	**9.44E+00**	**1.82E+00**
F20	1.19E+01	3.65E+00	1.50E+01	9.28E-07	1.47E+01	3.18E-01	**0.00E+00**	**0.00E+00**	**0.00E+00**	**0.00E+00**
F21	4.33E+02	2.21E+01	1.91E+03	1.78E+02	4.88E+02	4.33E+02	4.01E+02	1.77E-01	**4.00E+02**	**0.00E+00**
F22	4.42E+03	4.00E+02	9.71E+03	3.56E+02	**1.46E+03**	**6.02E+02**	6.95E+03	3.26E+02	6.05E+03	4.68E+02
F23	7.68E+03	3.87E+02	9.38E+03	3.92E+02	**6.34E+03**	**1.77E+03**	7.37E+03	2.85E+02	6.92E+03	2.13E+02
F24	2.16E+02	1.80E+01	5.32E+02	1.07E+02	2.77E+02	3.47E+01	2.00E+02	8.98E-02	**2.00E+02**	**7.65E-03**
F25	2.93E+02	2.37E+01	4.39E+02	1.86E+01	3.24E+02	4.11E+01	**2.29E+02**	**2.66E+01**	2.42E+02	3.20E+01
F26	**2.02E+02**	**2.51E+00**	5.14E+02	1.16E+02	3.68E+02	5.87E+01	2.14E+02	3.38E+01	2.58E+02	3.83E+01
F27	6.96E+02	2.09E+02	3.67E+03	2.69E+02	**1.13E+03**	**3.21E+02**	1.62E+03	2.91E+00	1.61E+03	3.89E-01
F28	2.26E+03	5.99E+02	6.38E+03	9.85E+02	3.62E+03	2.11E+03	9.90E+02	1.90E+01	**8.25E+02**	**1.85E+01**

**Table 8:** Wilcoxon rank sum test between IPRO and each comparison algorithm on CEC2013 test suite.

Function	p-value(vs.IPRO)			
	MSMPSO	MSCA	AND-RSN-PSO	IATTP
F1	0.000(-)	0.000(-)	0.000(-)	0.000(-)
F2	0.000(-)	0.000(-)	0.000(-)	0.000(-)
F3	1.000 (=)	0.000(-)	1.000 (=)	1.000 (=)
F4	0.049(-)	0.000(-)	0.000(-)	0.000(+)
F5	0.000(-)	0.000(-)	0.000(-)	0.000(-)
F6	0.000(-)	0.000(-)	0.000(-)	0.000(-)
F7	0.011(-)	0.000(-)	0.000(-)	1.000 (=)
F8	0.716 (=)	0.000(-)	0.000(-)	0.716 (=)
F9	1.000 (=)	0.000(-)	0.000(-)	1.000 (=)
F10	0.000(-)	0.000(-)	0.000(-)	0.000(-)
F11	0.082 (=)	0.000(-)	1.000 (=)	1.000 (=)
F12	0.000(-)	0.000(-)	0.000(-)	1.000 (=)
F13	0.000(-)	0.000(-)	0.000(-)	1.000 (=)
F14	0.000(-)	0.000(-)	0.000(-)	0.994 (=)
F15	0.000(-)	0.000(-)	0.000(-)	0.000(+)
F16	0.214 (=)	0.000(-)	0.028(-)	0.367 (=)
F17	0.000(-)	0.000(-)	0.000(+)	0.000(-)
F18	0.000(-)	0.000(-)	0.000(-)	0.000(-)
F19	0.000(-)	0.000(-)	0.000(-)	0.000(-)
F20	0.000(-)	0.000(-)	0.000(-)	1.000 (=)
F21	0.000(-)	0.000(-)	0.001(-)	0.000(-)
F22	0.000(+)	0.000(-)	0.000(+)	0.000(-)
F23	0.000(-)	0.000(-)	0.000(+)	0.000(-)
F24	0.000(-)	0.000(-)	0.000(-)	0.161 (=)
F25	0.000(-)	0.000(-)	0.000(-)	0.385 (=)
F26	0.000(+)	0.000(-)	0.000(-)	0.000(+)
F27	0.000(-)	0.000(-)	0.000(+)	0.000(-)
F28	0.000(-)	0.000(-)	0.000(-)	0.000(-)
+/ = /-	2/5/21	0/0/28	4/2/22	3/12/13

**Table 9:** Friedman- test of 5 algorithms (D = 30).

	MSMPSO	MSCA	AND-RSN-PSO	IATTP	IPRO
*Avg*.*rank*	2.946	4.964	3.089	2.196	1.804
*sort*	3	5	4	2	1

Table 7 shows that for the 30-dimensional optimization problems, MSCA does not obtain the global optimal value of any function, MSMPSO obtains the global optimal value on F3 and F9, AND-RSN-PSO obtains the global optimal value on F3 and F11, IATTP obtains the global optimal value on 7 functions (including. F3, F7, F9, F11, F12, F13, and F20), and our proposed IPRO obtains the global optimal value on 9 functions (including F1, F2, F3, F5, F7, F9, F11, F12, F13, and F20). In addition, the number of best results obtained by MSMPSO, MSCA, AND-RSN-PSO, IATTP, and IPRO were 4, 0, 6, 11, and 18, respectively. Meanwhile, Table 8 shows that compared with MSMPSO, IPRO demonstrates a similar performance on 5 functions (F3, F8, F9, F11, and F16), a significantly inferior performance on F22 and F26, and a significantly better performance on 21 functions. IPRO significantly outperformed MSCA on all 28 functions, but compared with AND-RSN-PSO, IPRO demonstrated a significantly worse performance on 4 functions (including. F17, F22, F23, and F27), a similar performance on 2 functions (including F3 and F11), and a significantly better performance on 22 functions. Compared with IATTP, IPRO demonstrated a significantly worse performance on 3 functions (including F4, F15, and F26), a similar performance on 12 functions, and a significantly better performance on 13 functions. Table 9 shows that among the above five algorithms, MSCA demonstrates the poorest performance, AND-RSN-PSO and MSMPAO are slightly better than MSCA, IATTP is better than the three aforementioned algorithms, and our proposed IPRO demonstrates the best overall performance.

To compare the convergence speed of these algorithms more intuitively, Figs 1–28 illustrates the results of each algorithm running once randomly on each function. The horizontal and vertical coordinates of this figure represent the number of function evaluations and the logarithmic of the function value obtained by each algorithm in the corresponding function evaluation times, respectively.

**Fig 1:**
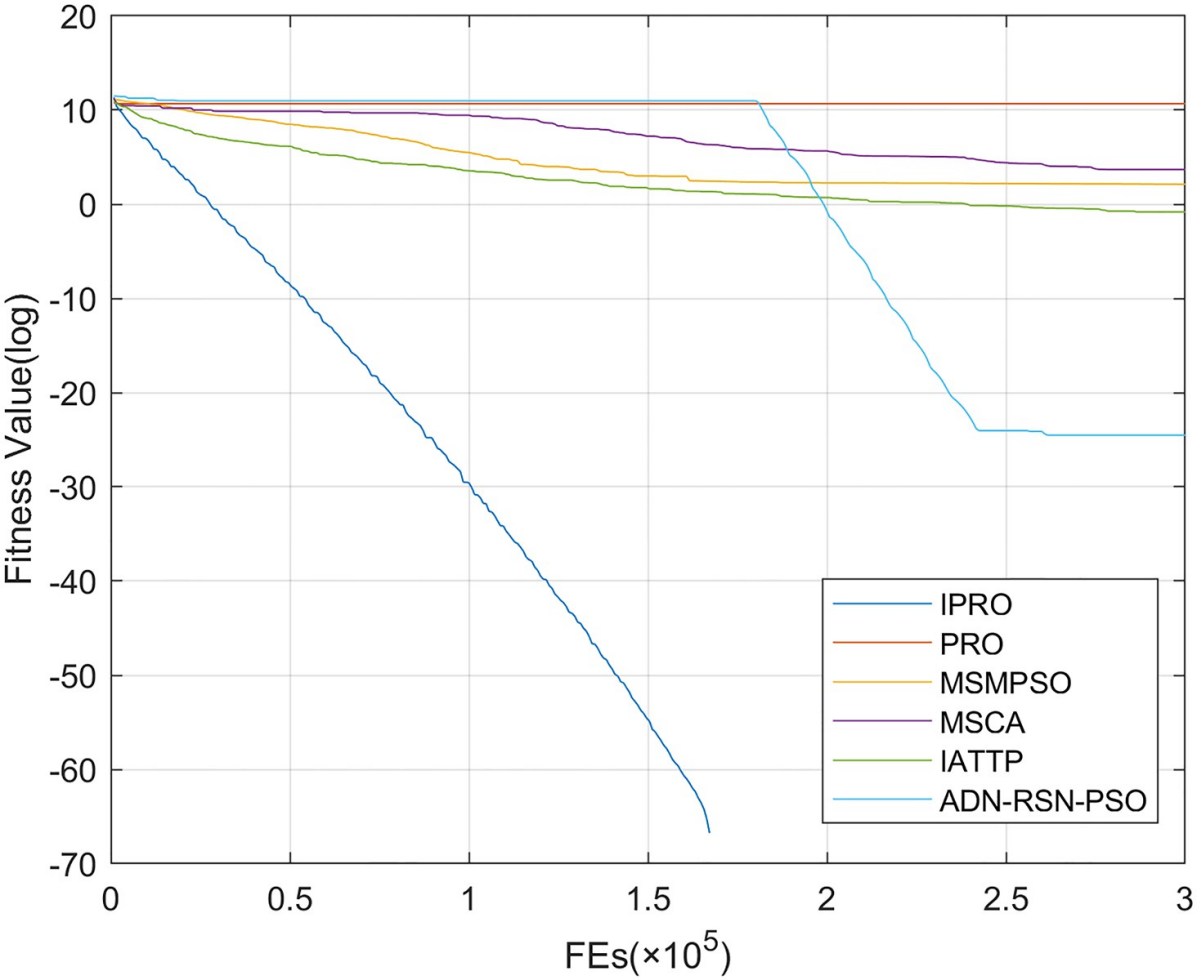
Convergence curves on F1.

**Fig 2:**
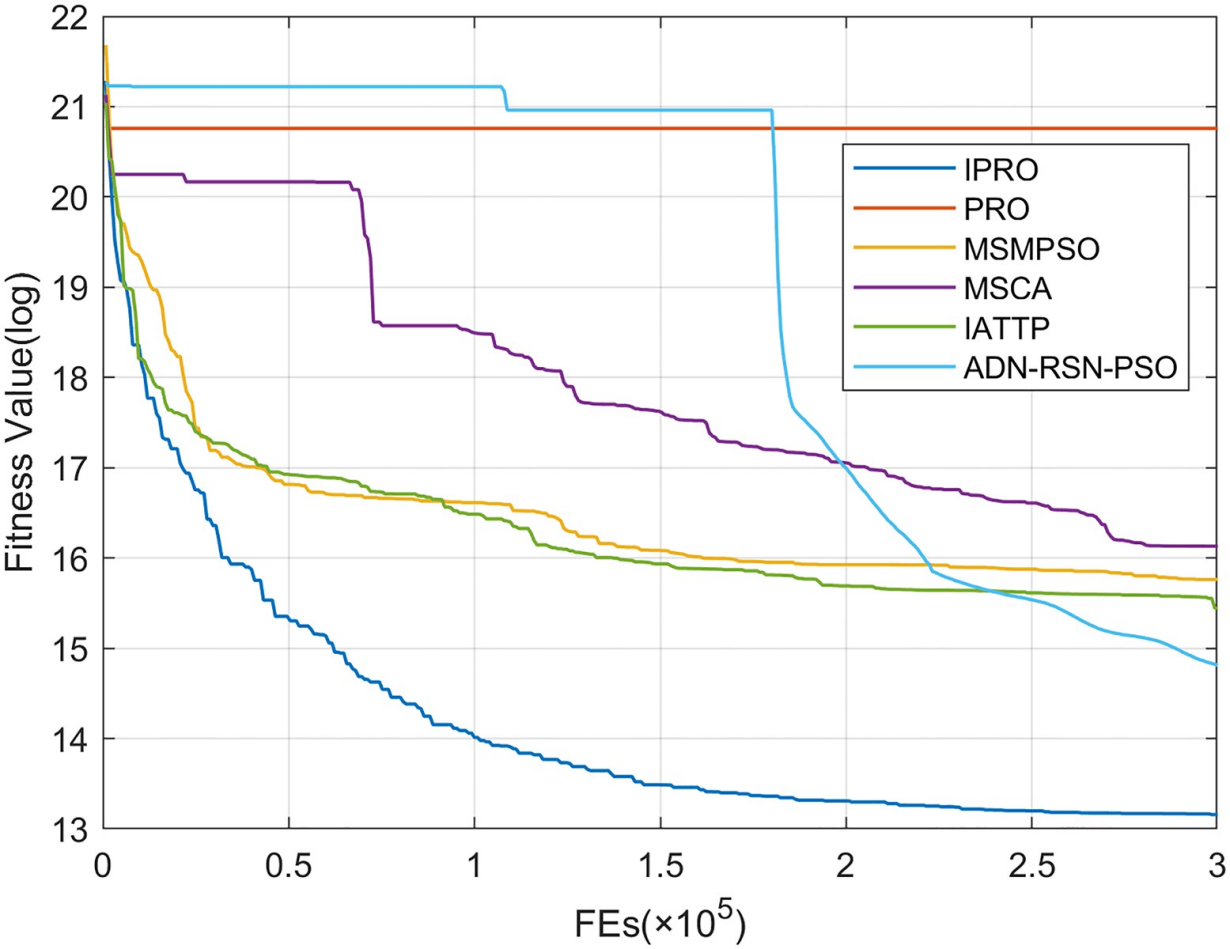
Convergence curves on F2.

**Fig 3:**
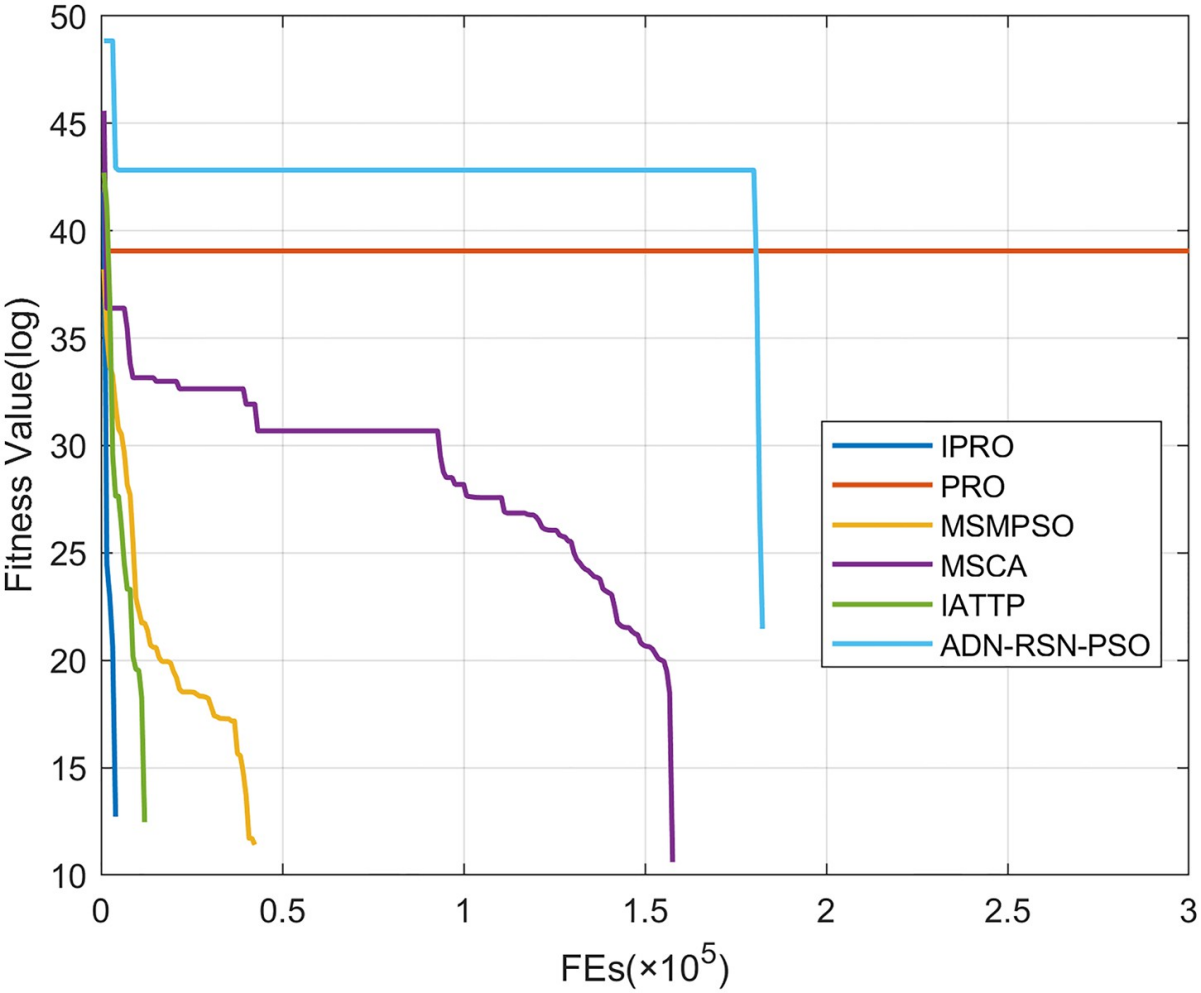
Convergence curves on F3.

**Fig 4:**
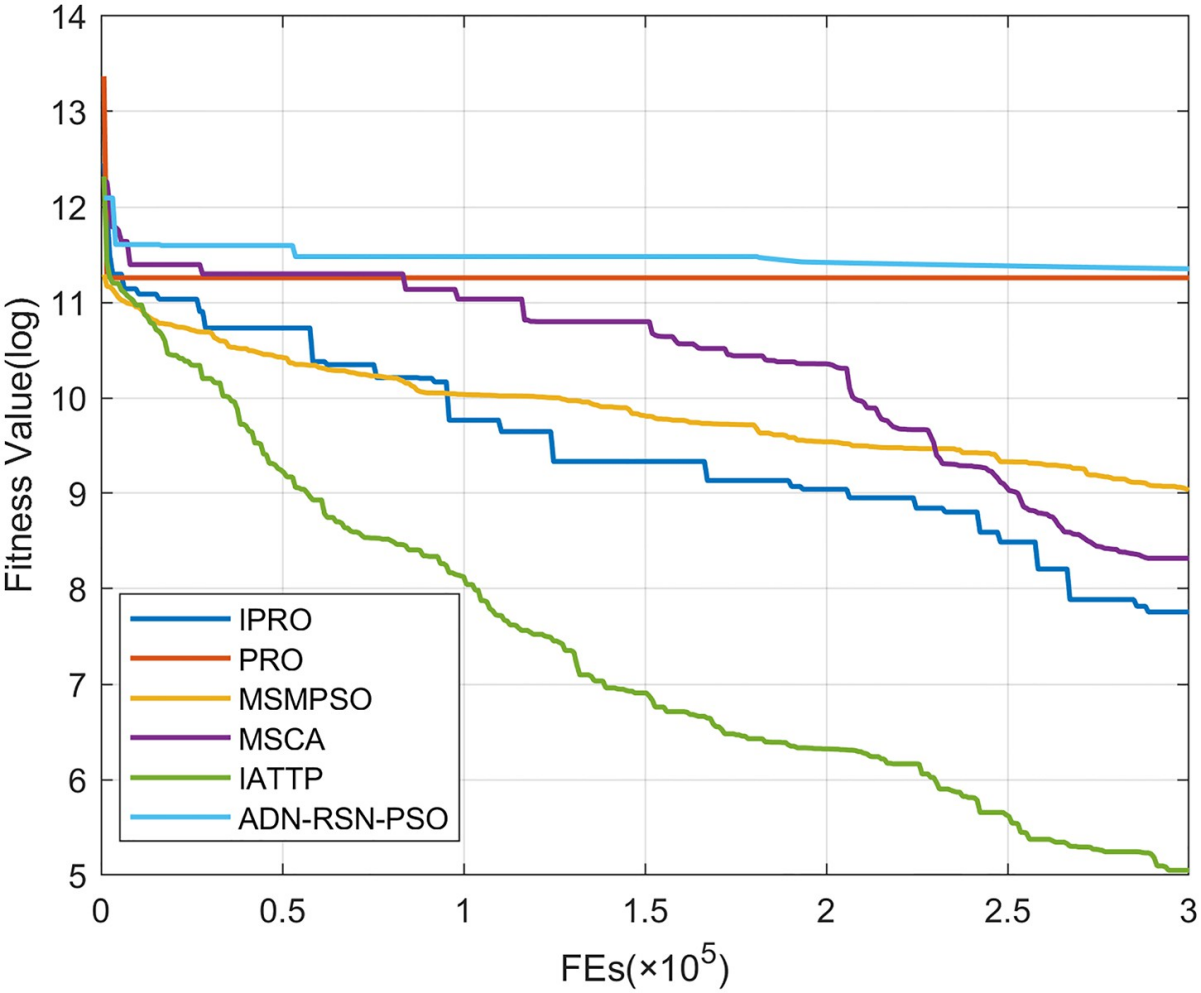
Convergence curves on F4.

**Fig 5:**
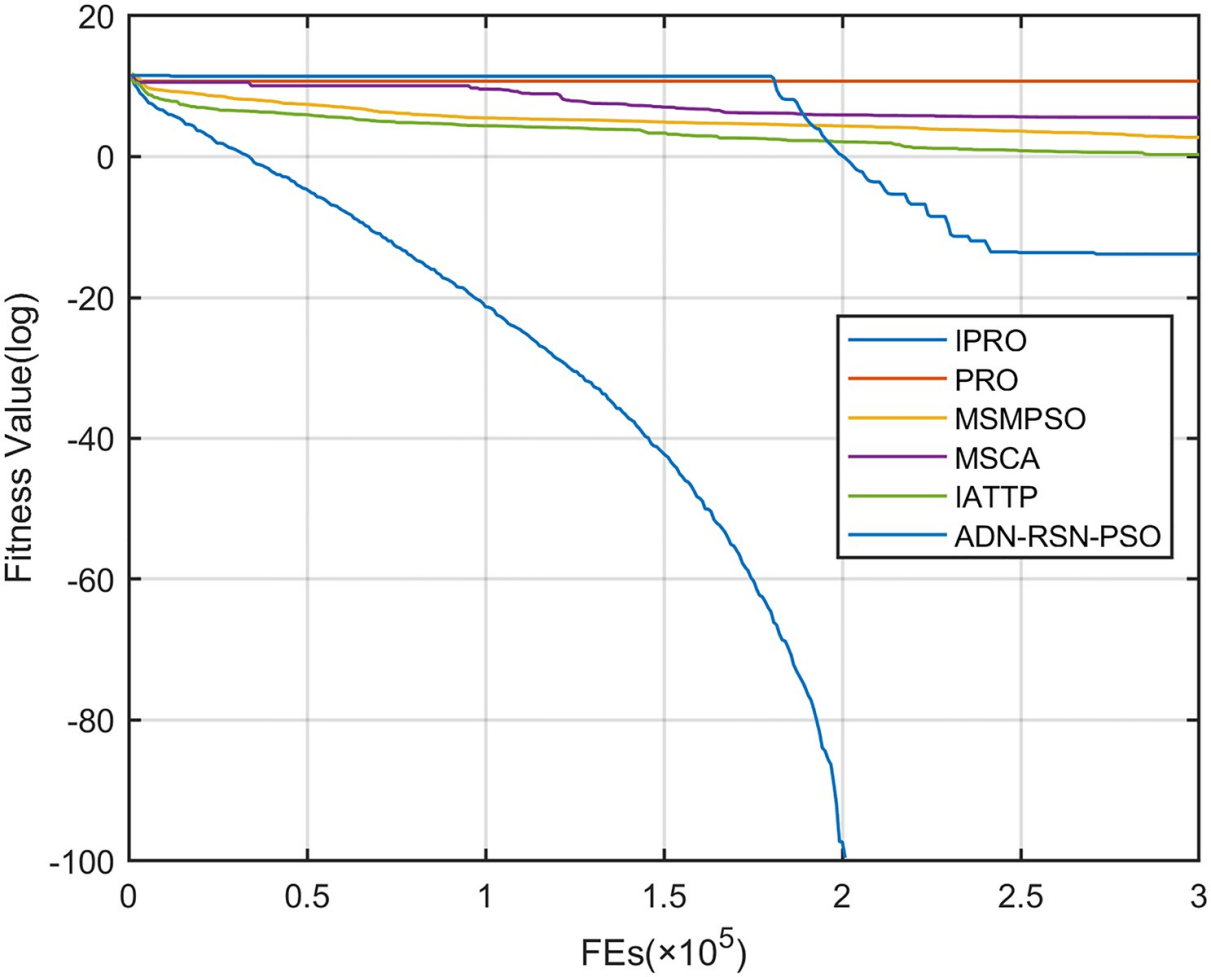
Convergence curves on F5.

**Fig 6:**
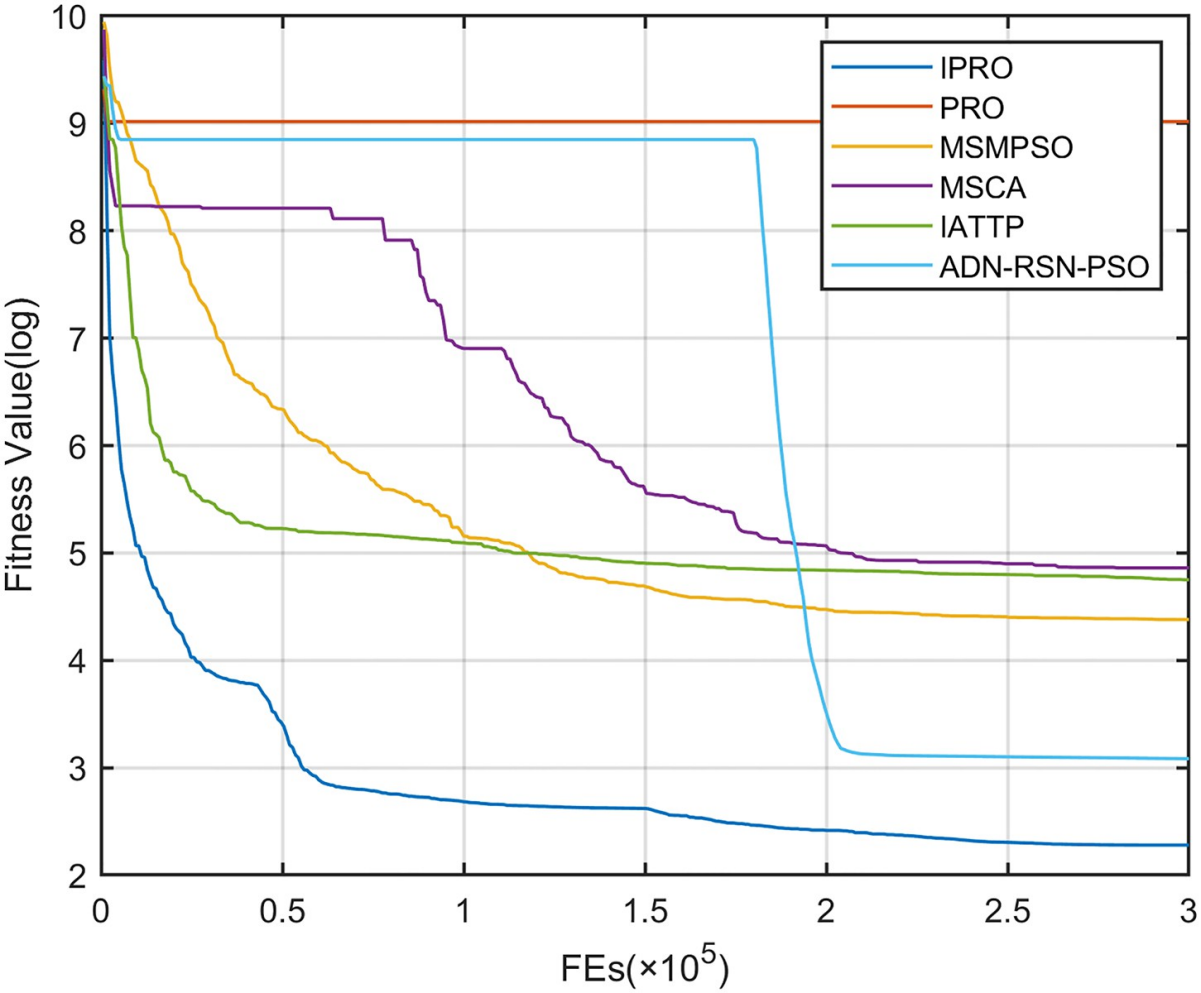
Convergence curves on F6.

**Fig 7:**
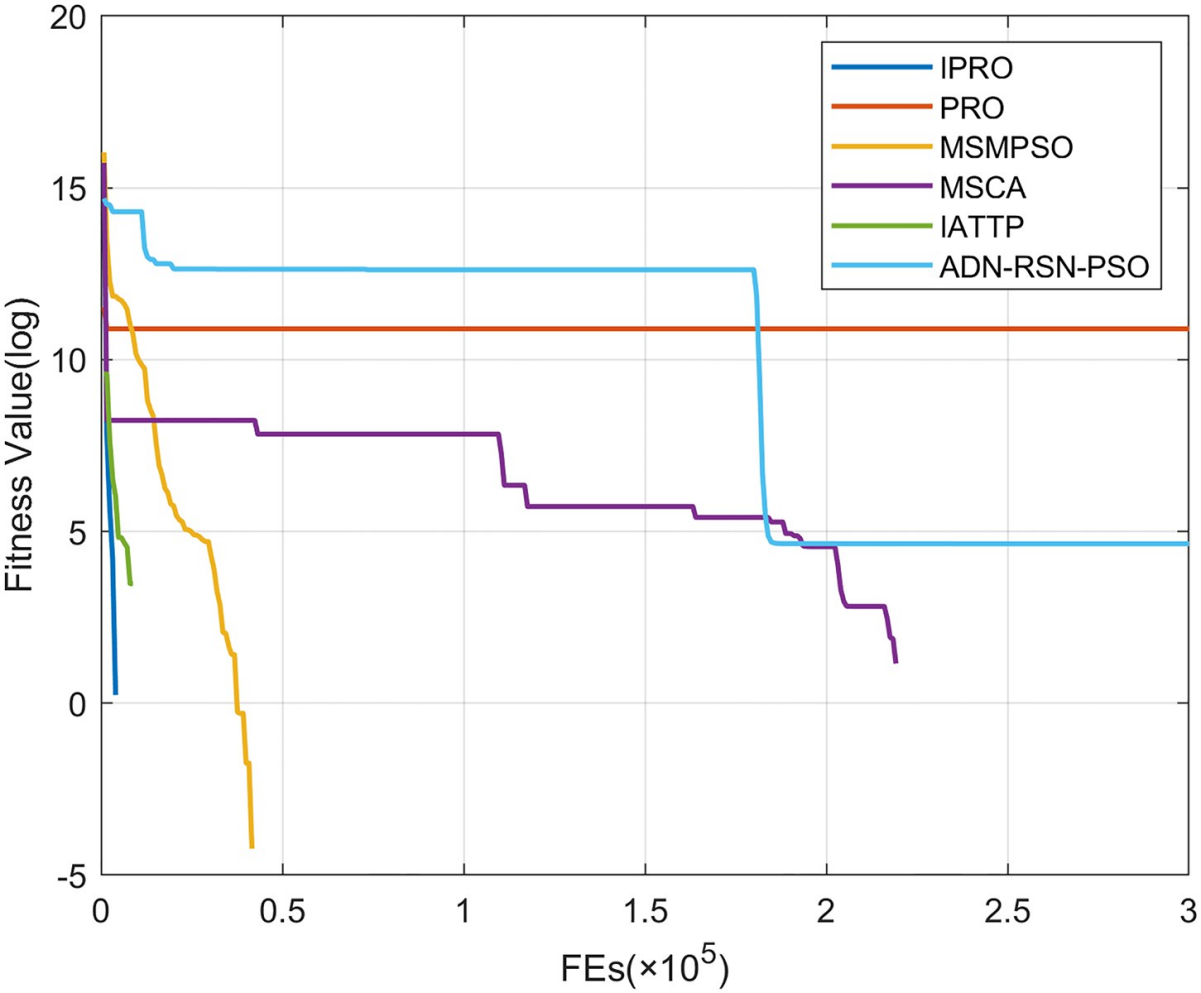
Convergence curves on F7.

**Fig 8:**
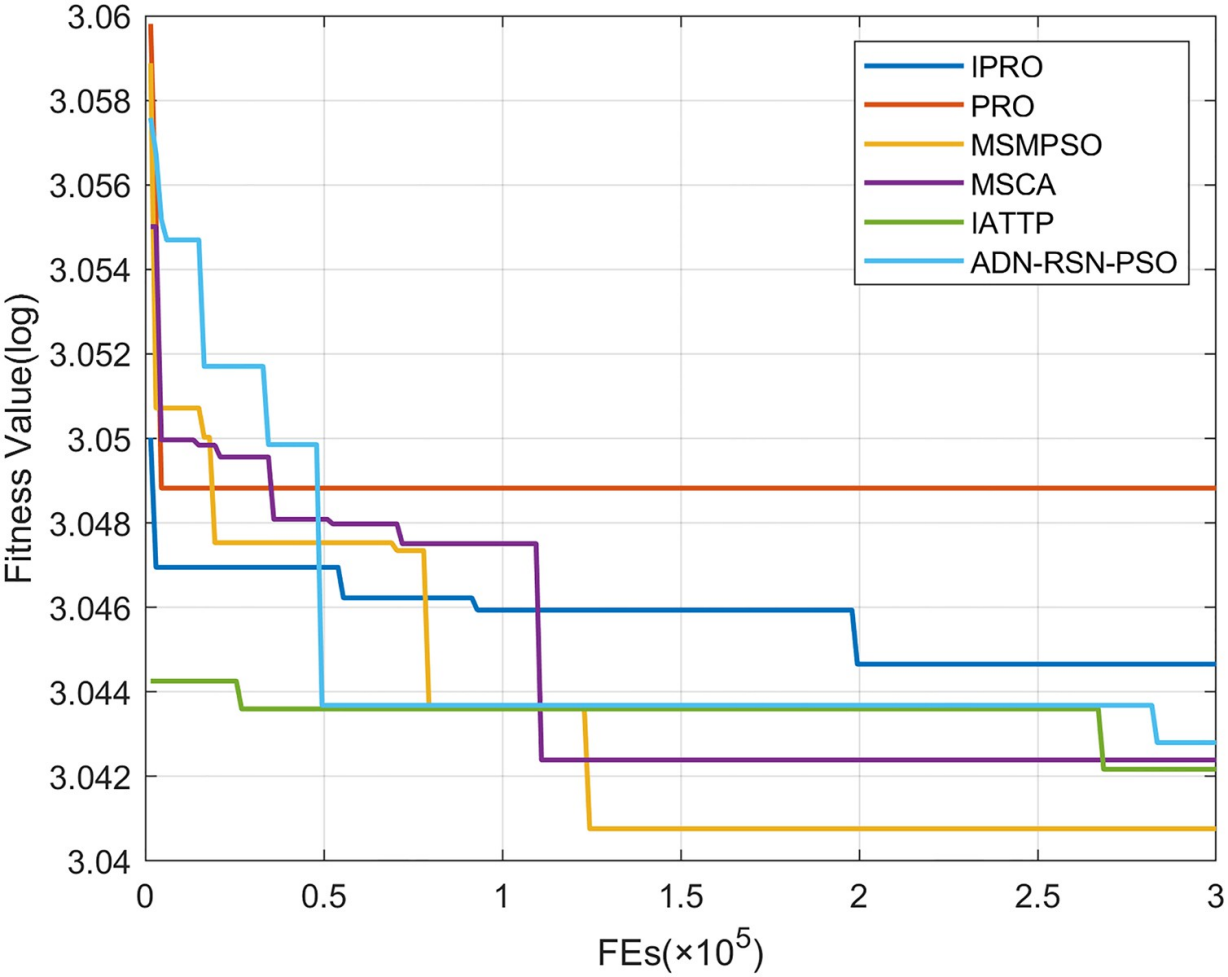
Convergence curves on F8.

**Fig 9:**
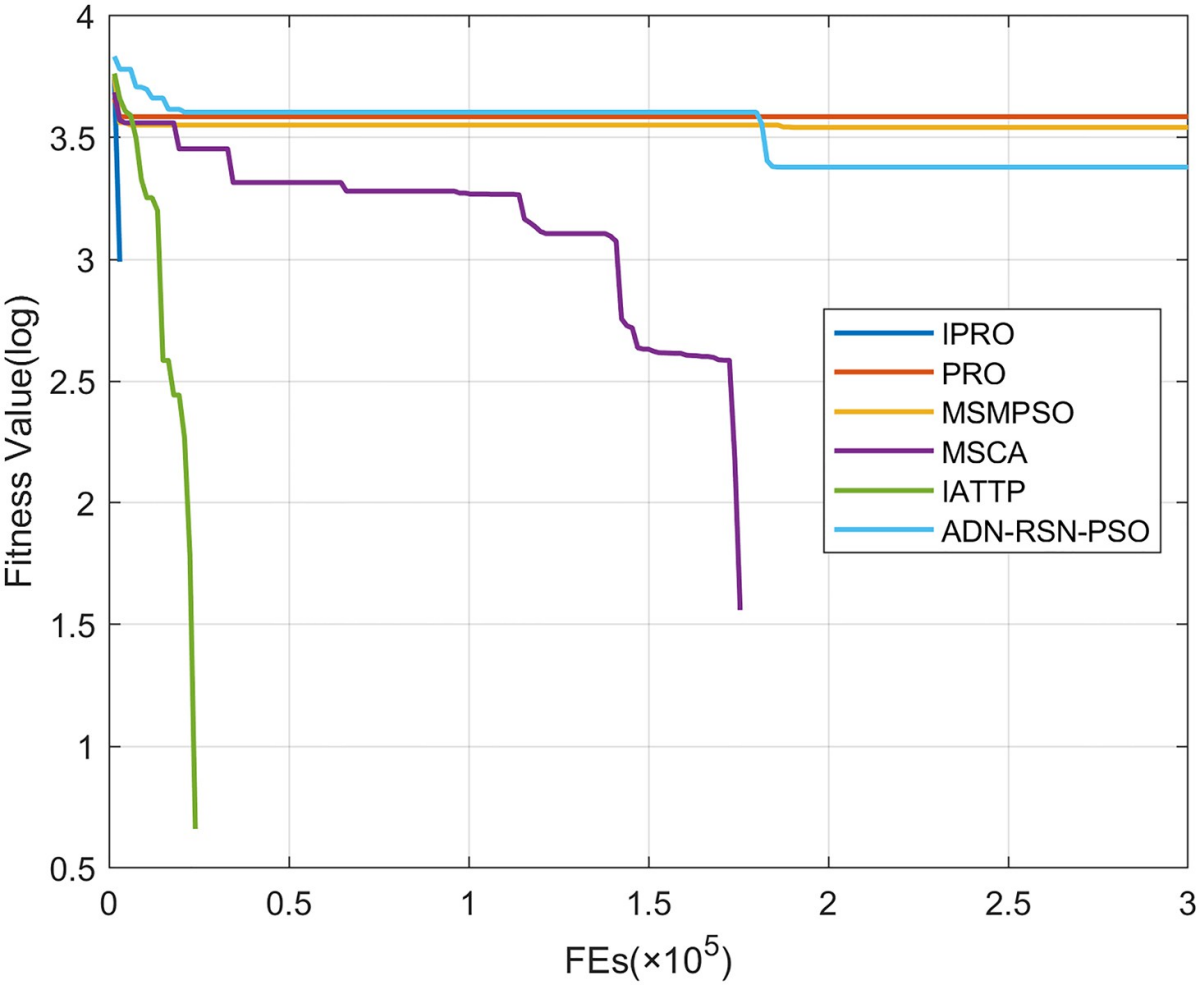
Convergence curves on F9.

**Fig 10:**
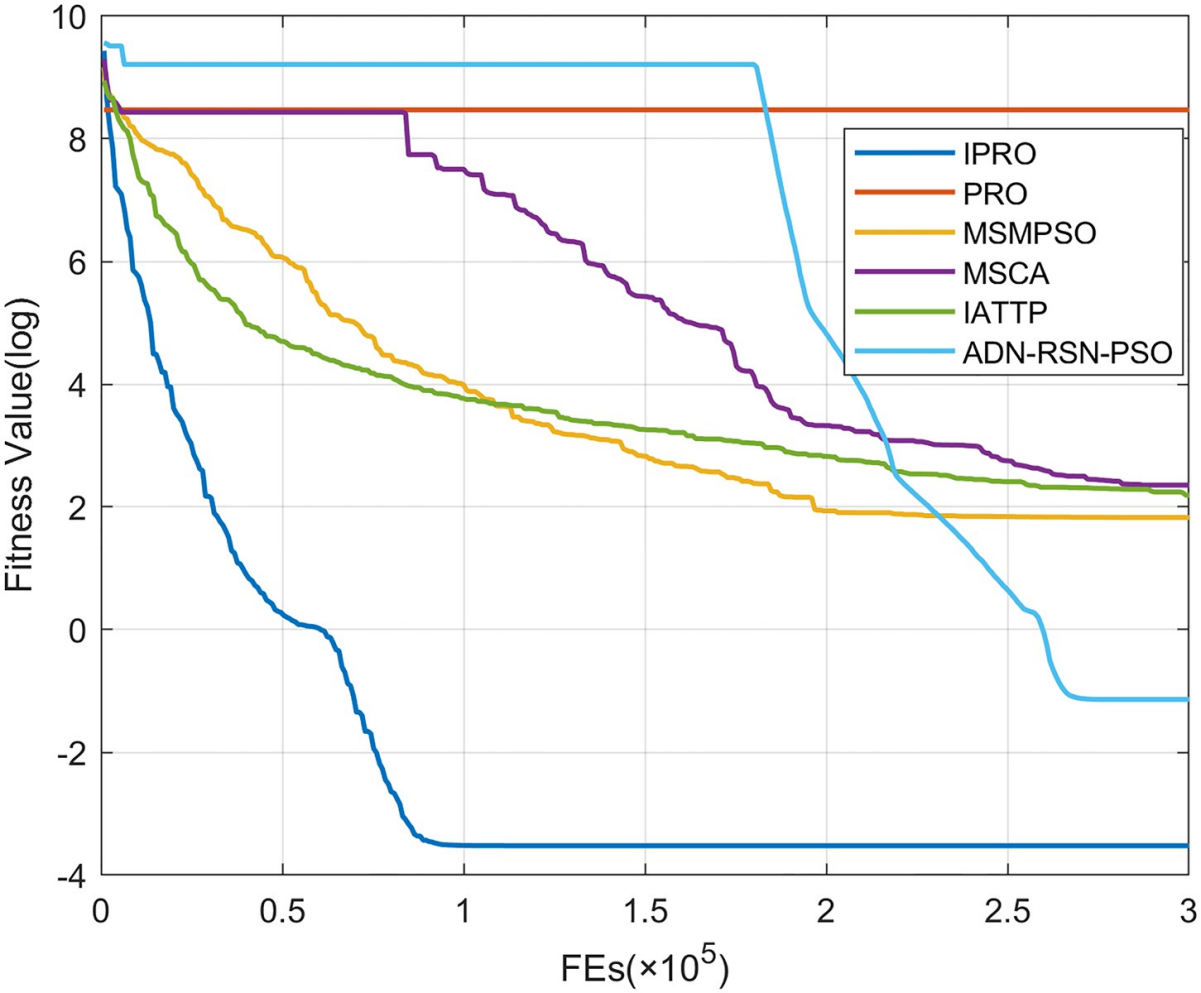
Convergence curves on F10.

**Fig 11:**
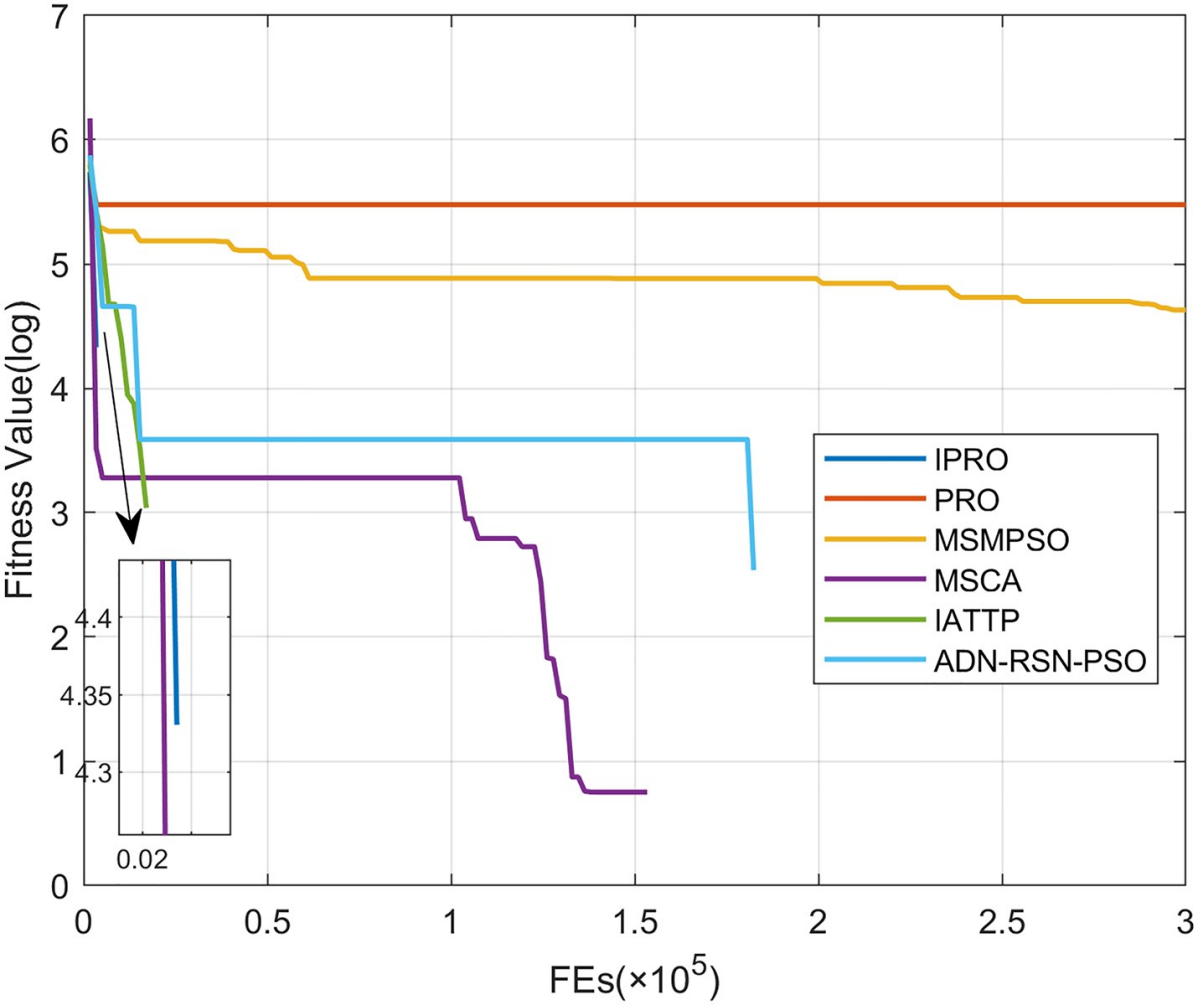
Convergence curves on F11.

**Fig 12:**
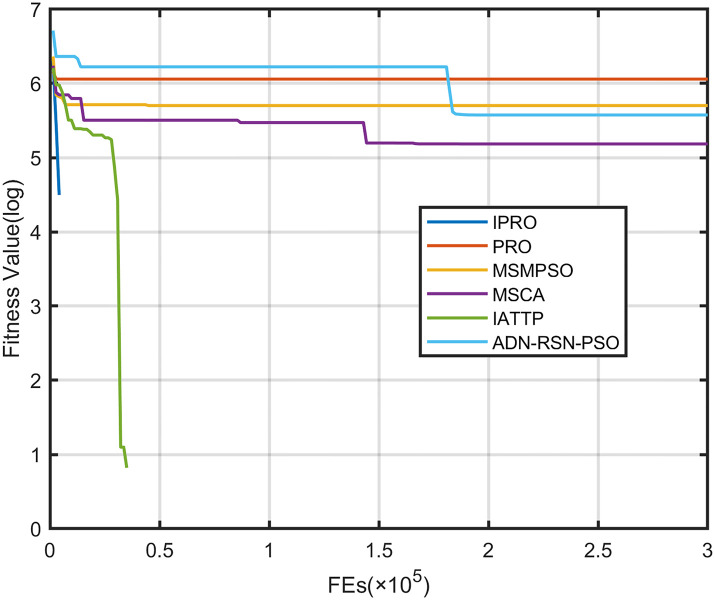
Convergence curves on F12.

**Fig 13:**
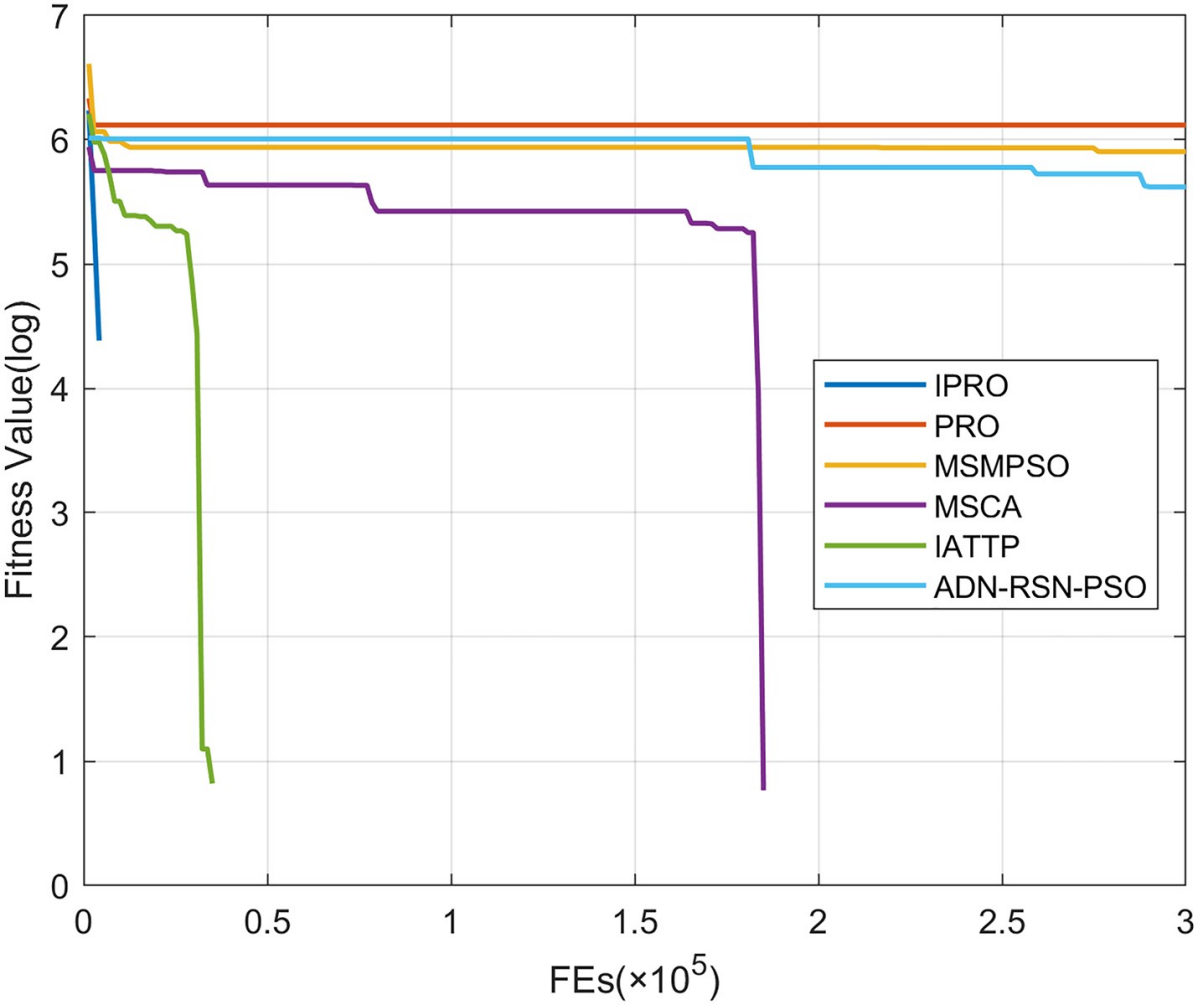
Convergence curves on F13.

**Fig 14:**
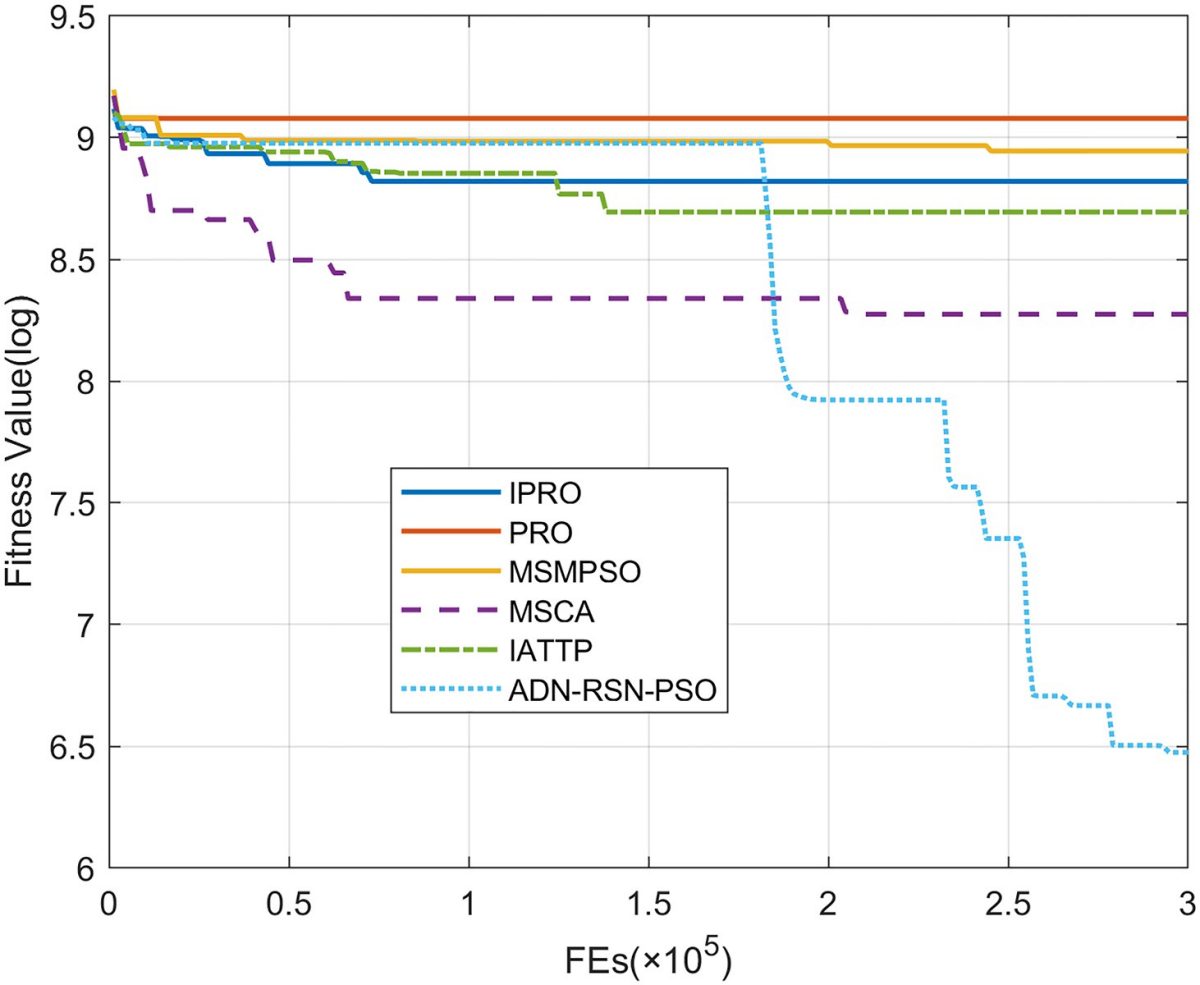
Convergence curves on F14.

**Fig 15:**
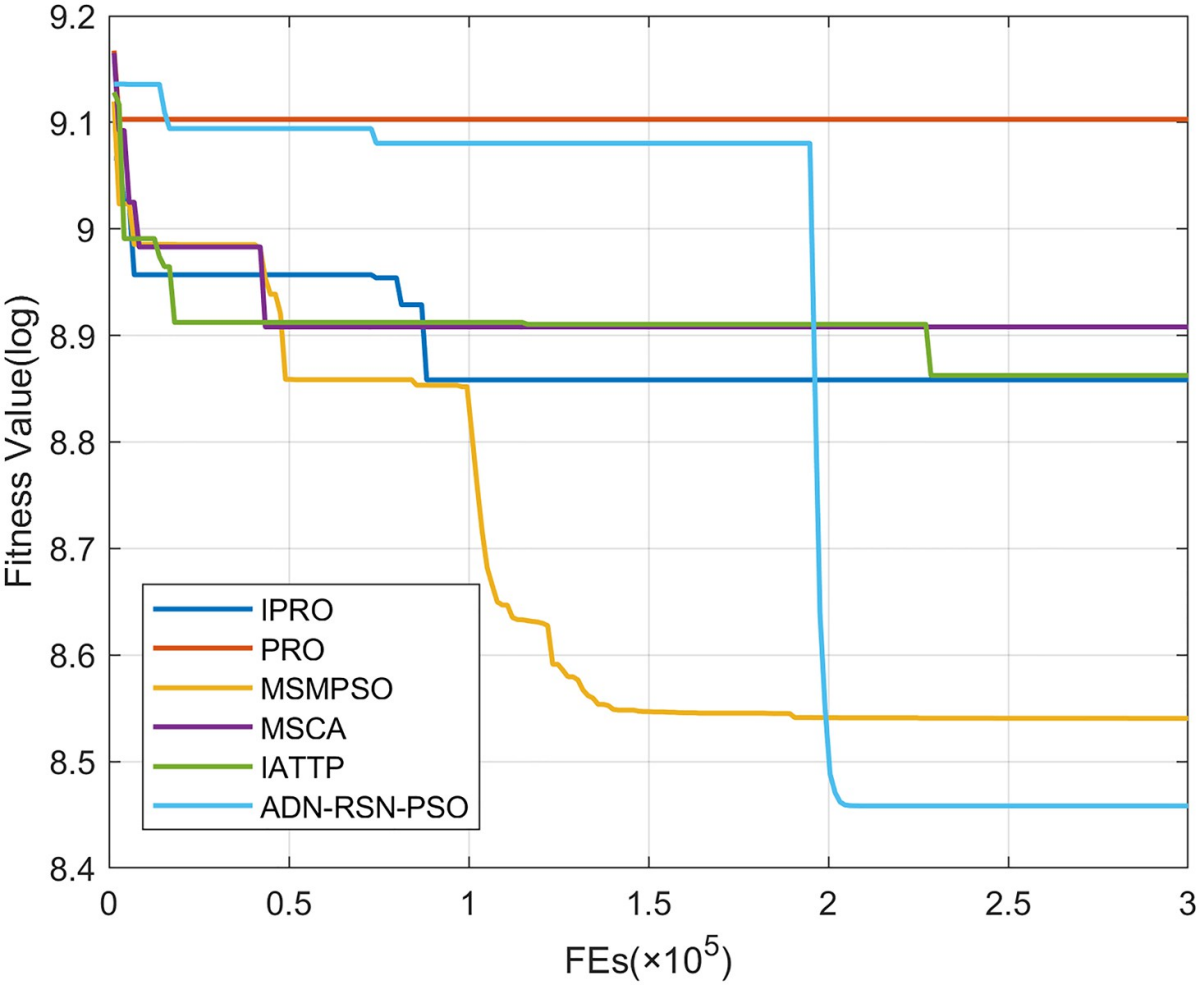
Convergence curves on F15.

**Fig 16:**
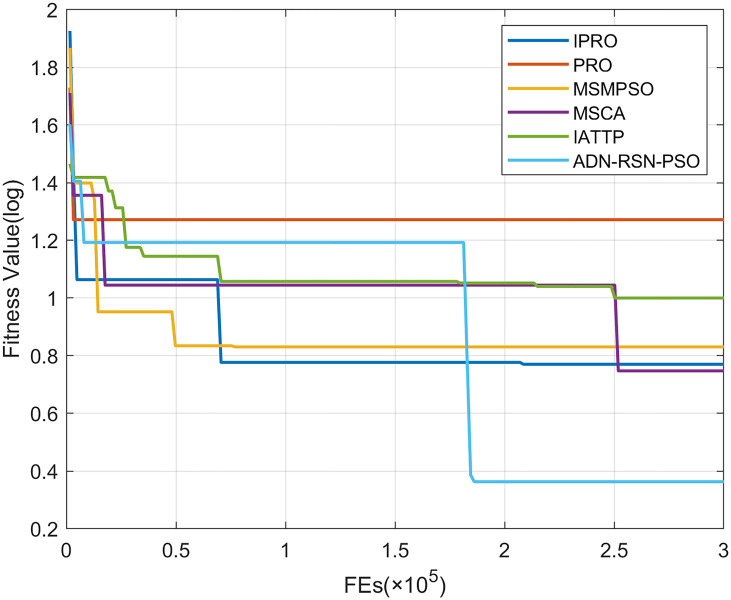
Convergence curves on F16.

**Fig 17:**
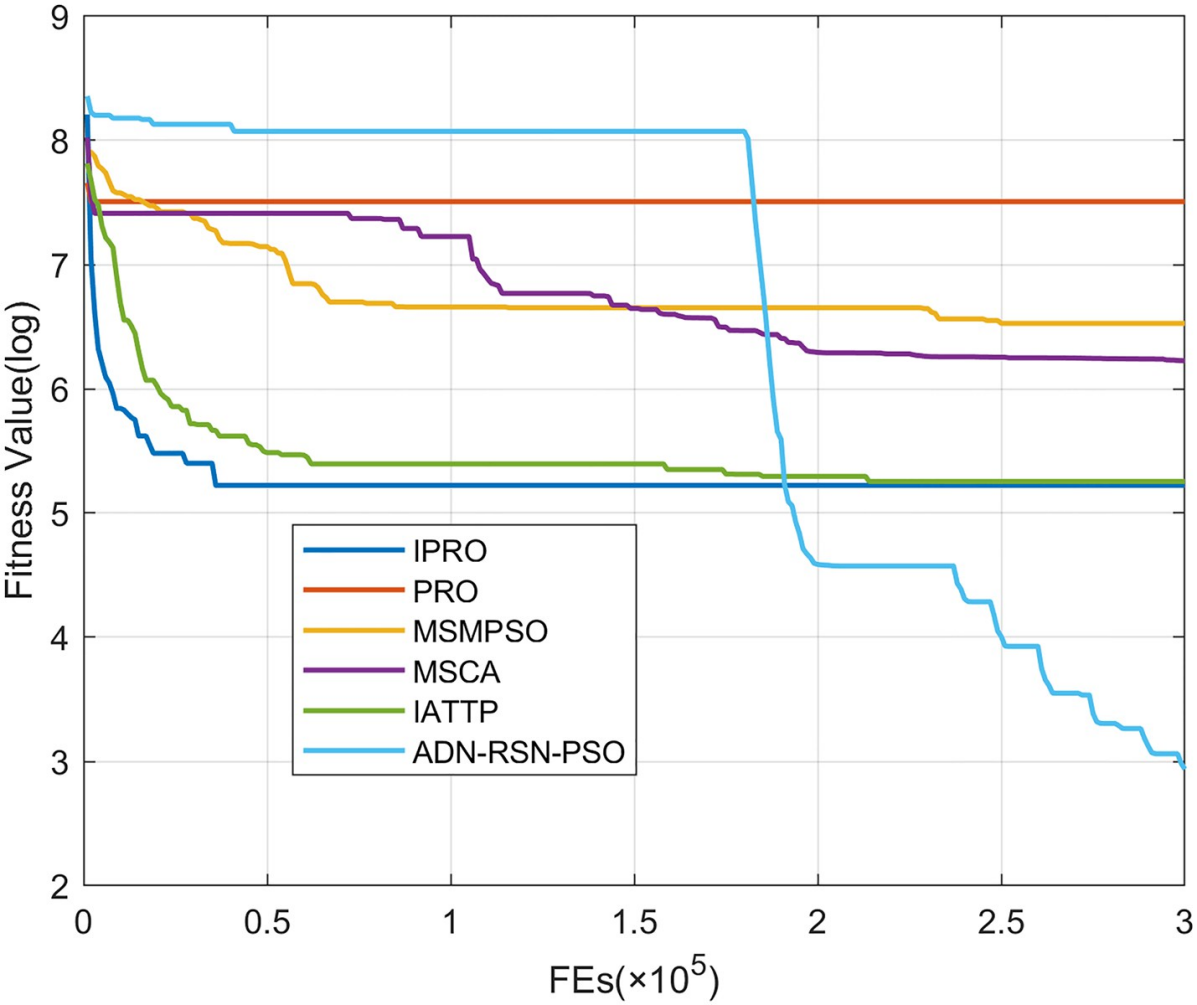
Convergence curves on F17.

**Fig 18:**
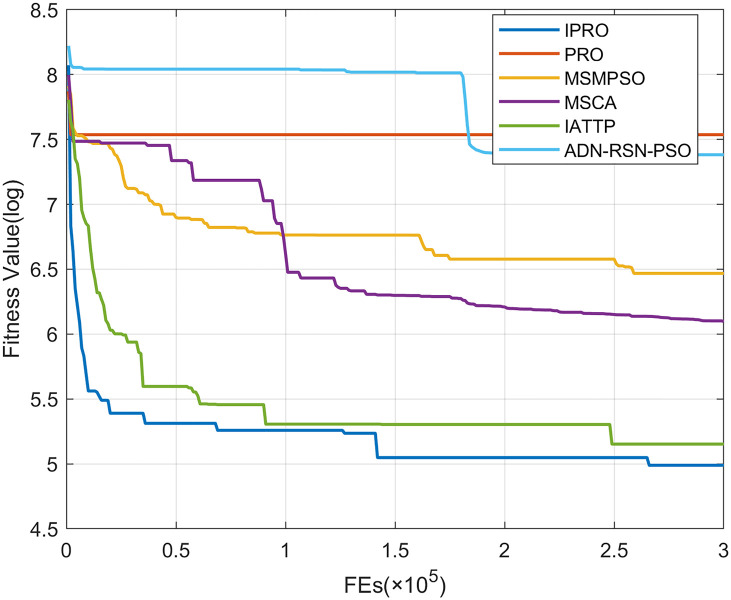
Convergence curves on F18.

**Fig 19:**
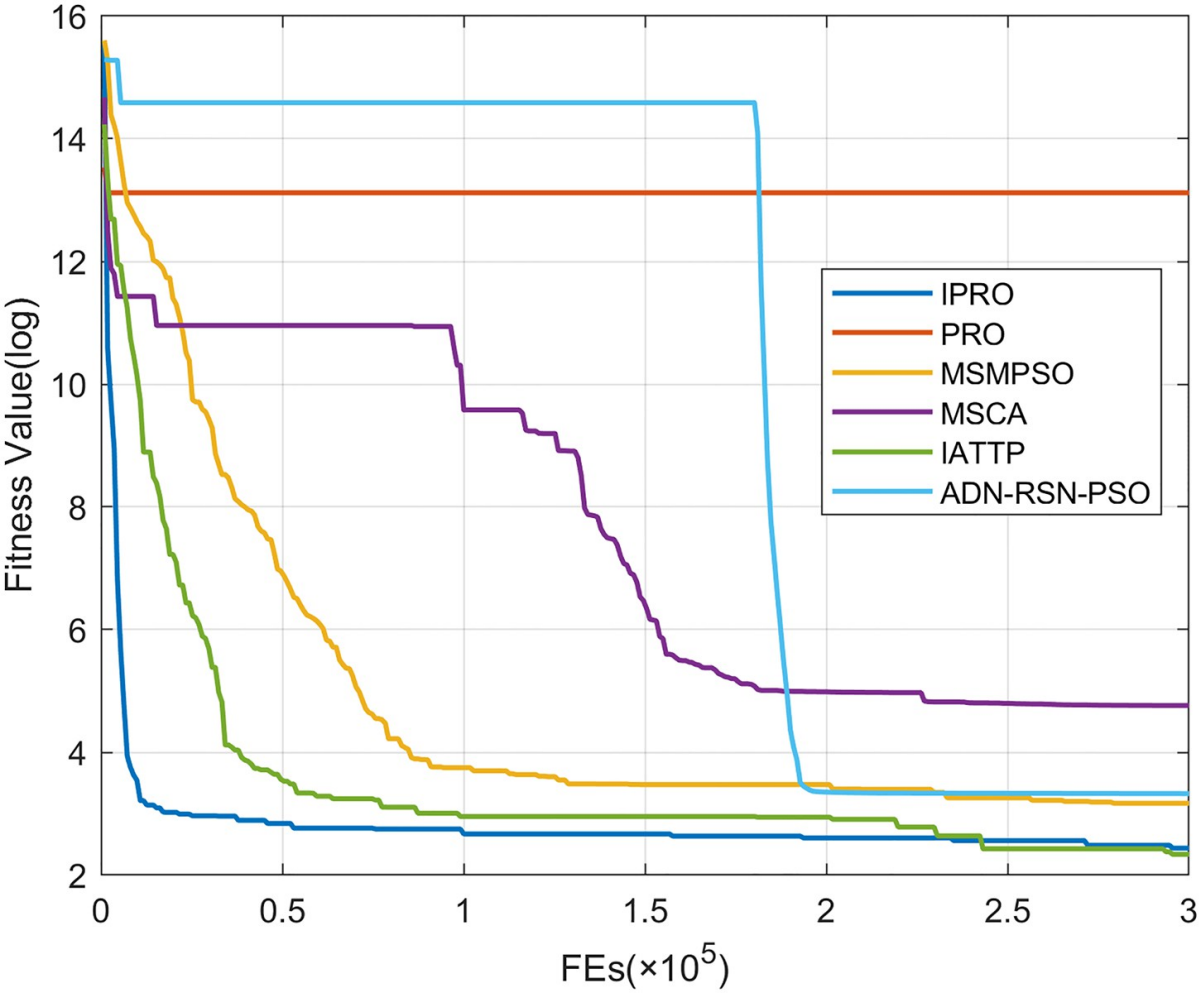
Convergence curves on F19.

**Fig 20:**
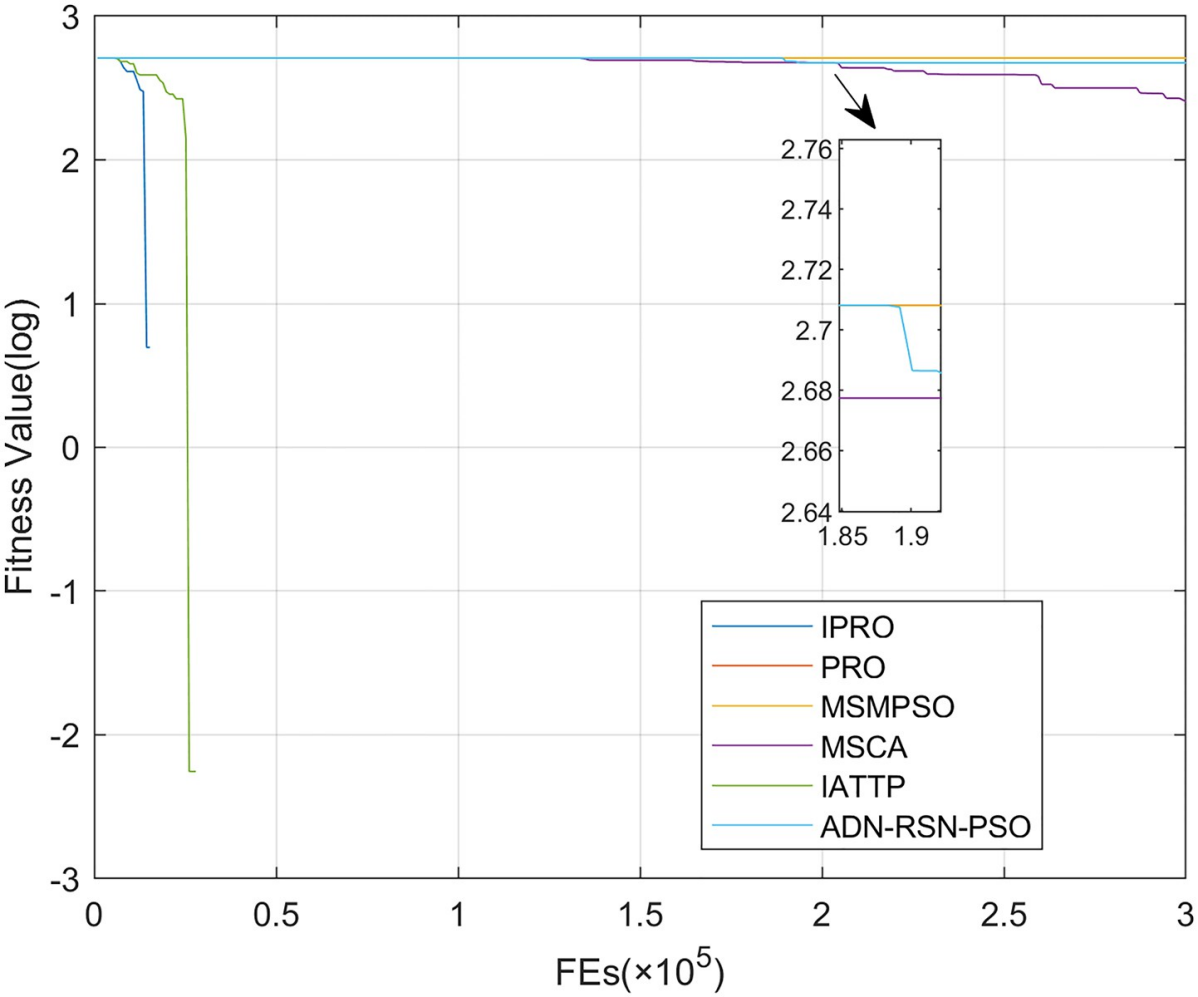
Convergence curves on F20.

**Fig 21:**
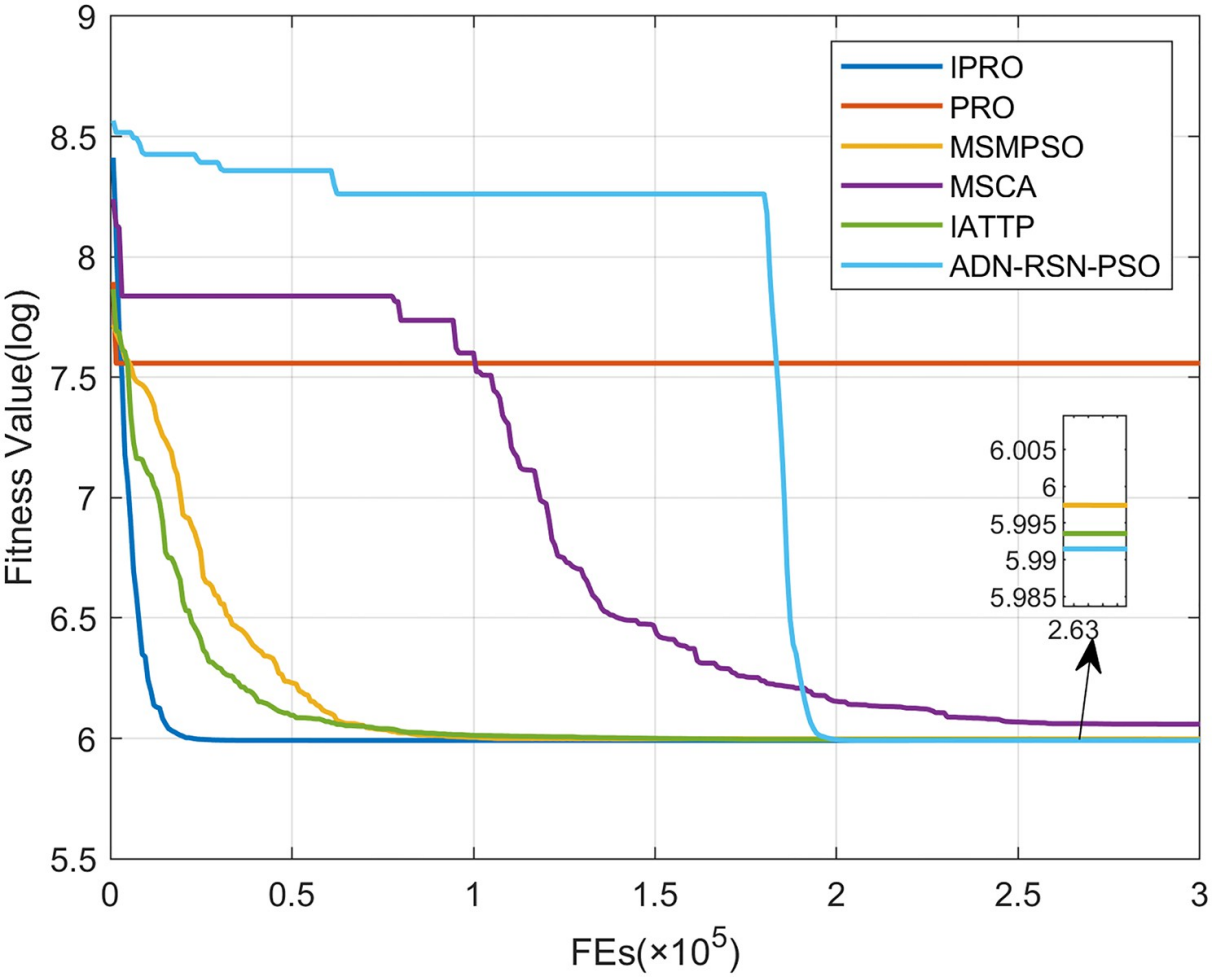
Convergence curves on F21.

**Fig 22:**
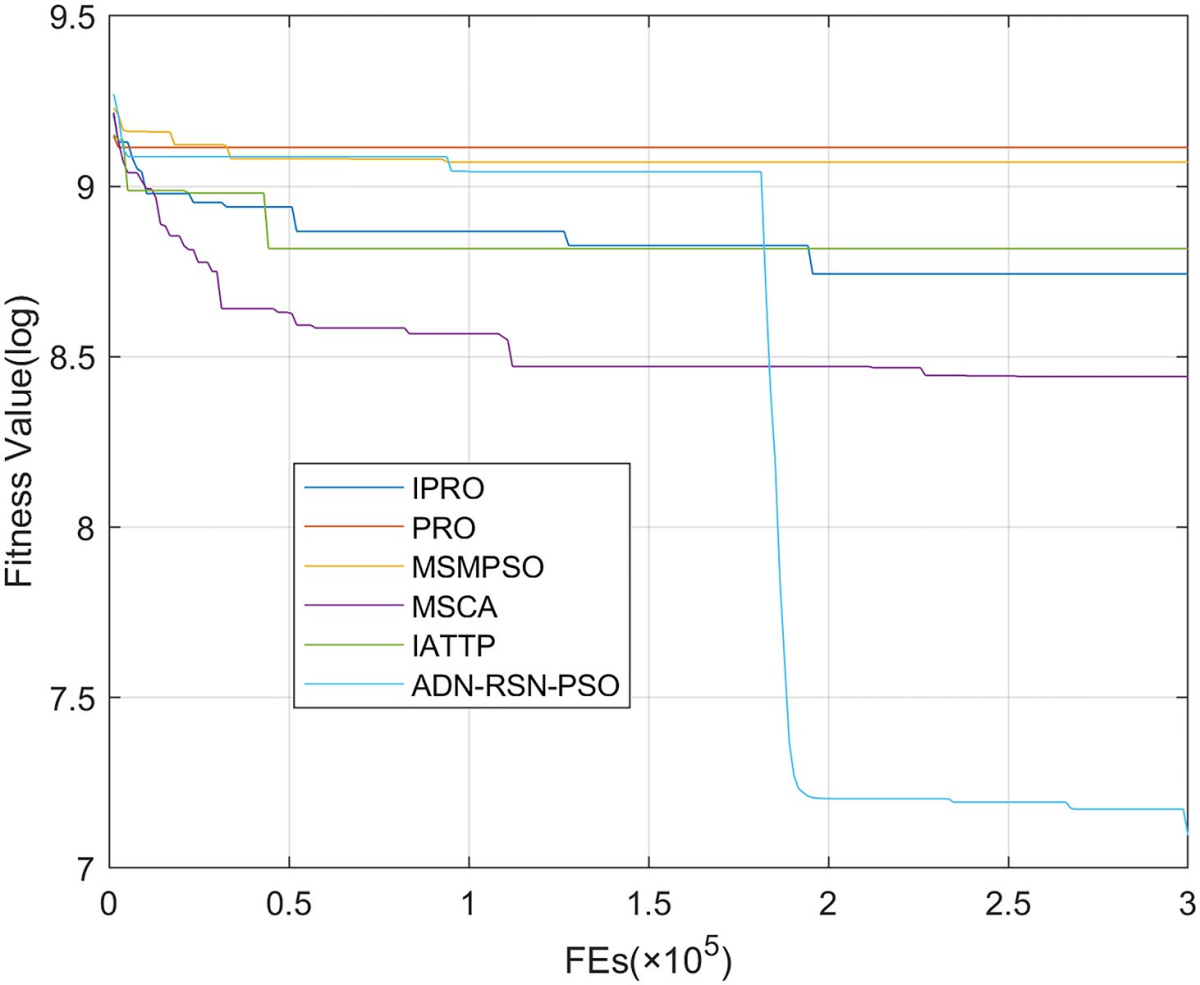
Convergence curves on F22.

**Fig 23:**
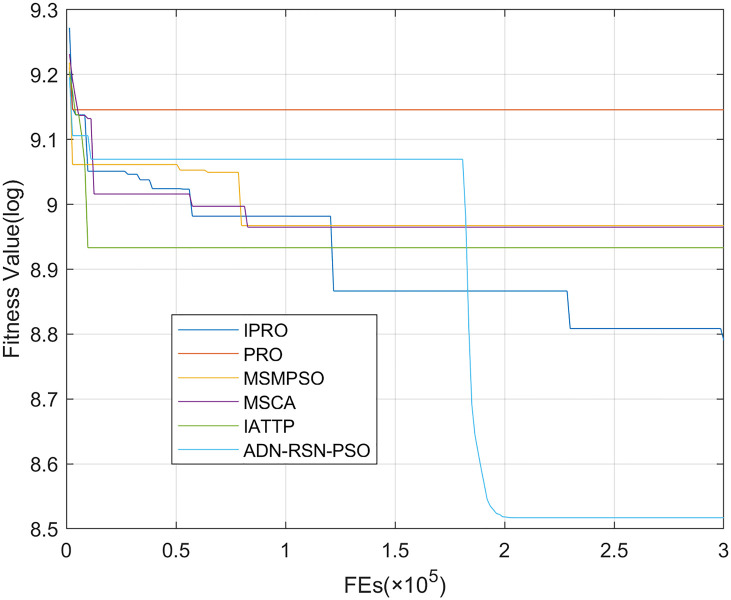
Convergence curves on F23.

**Fig 24:**
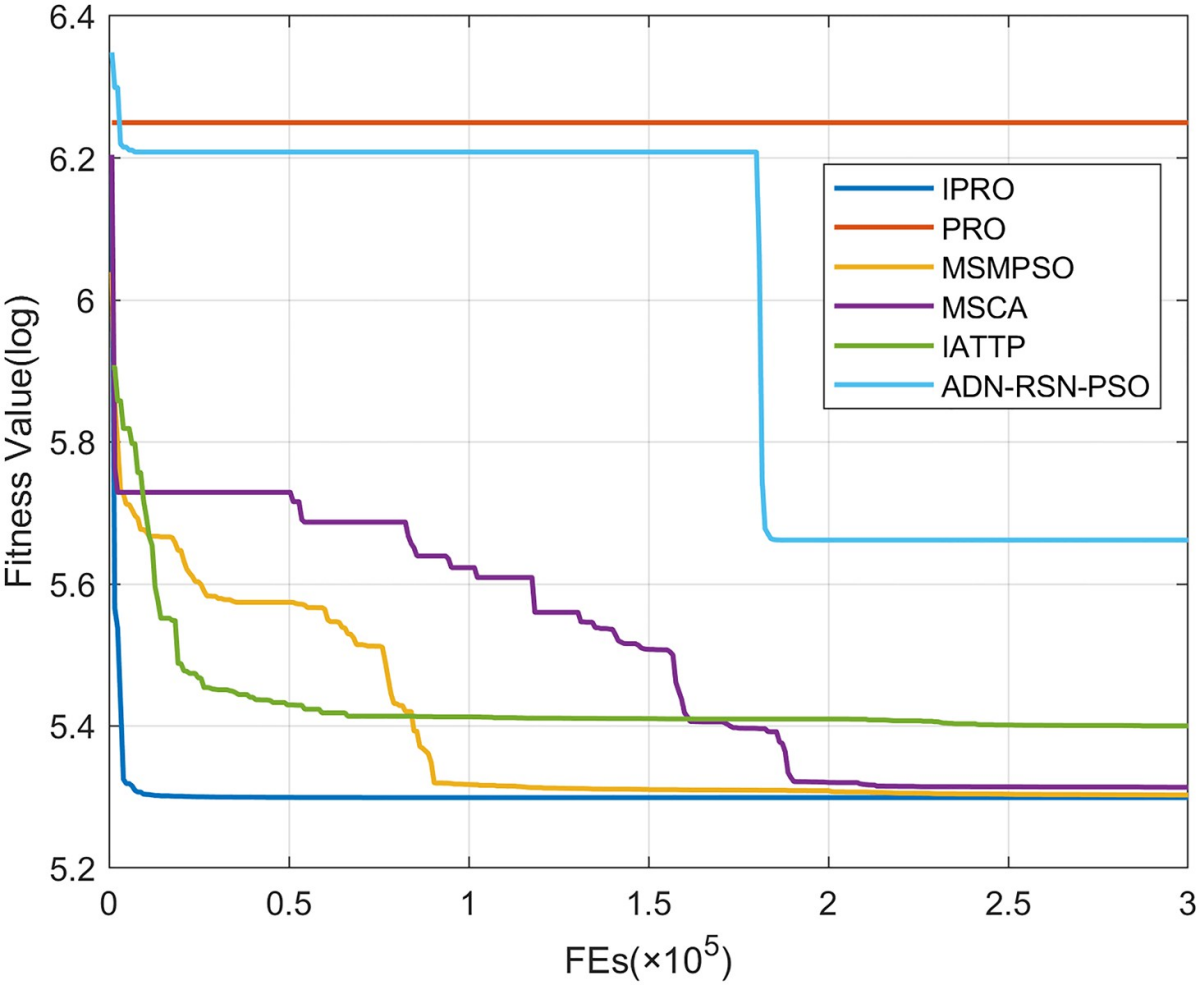
Convergence curves on F24.

**Fig 25:**
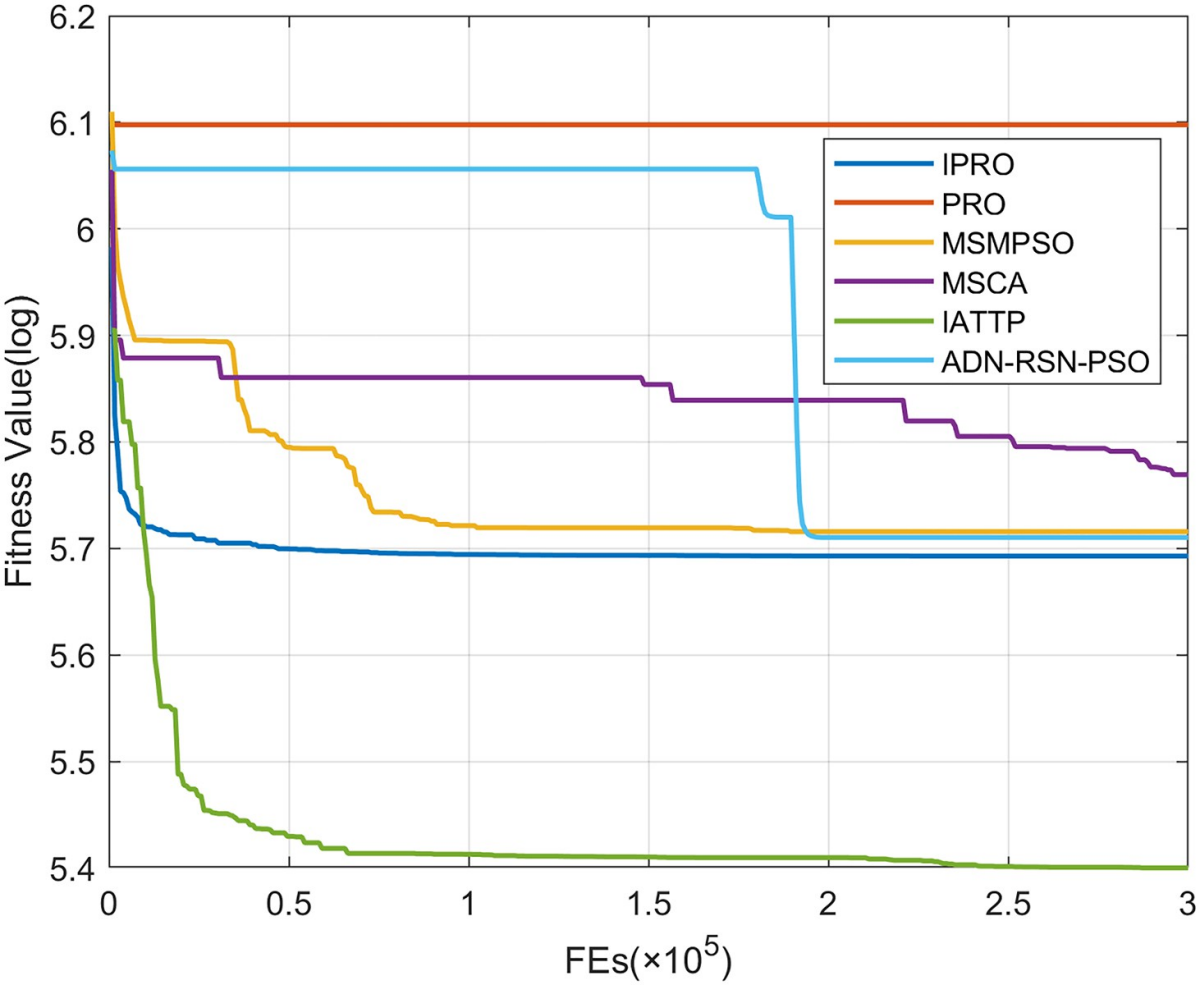
Convergence curves on F25.

**Fig 26:**
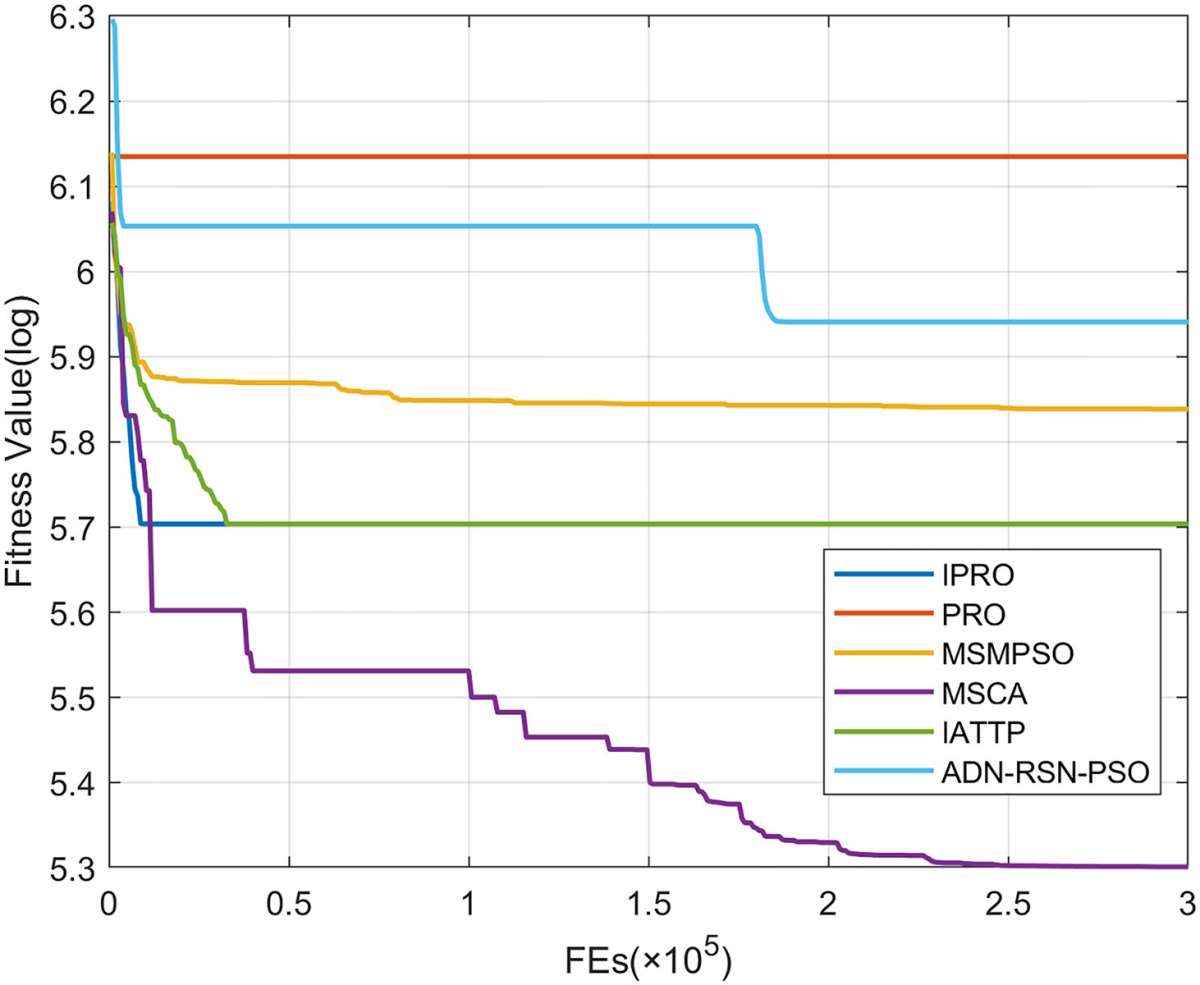
Convergence curves on F26.

**Fig 27:**
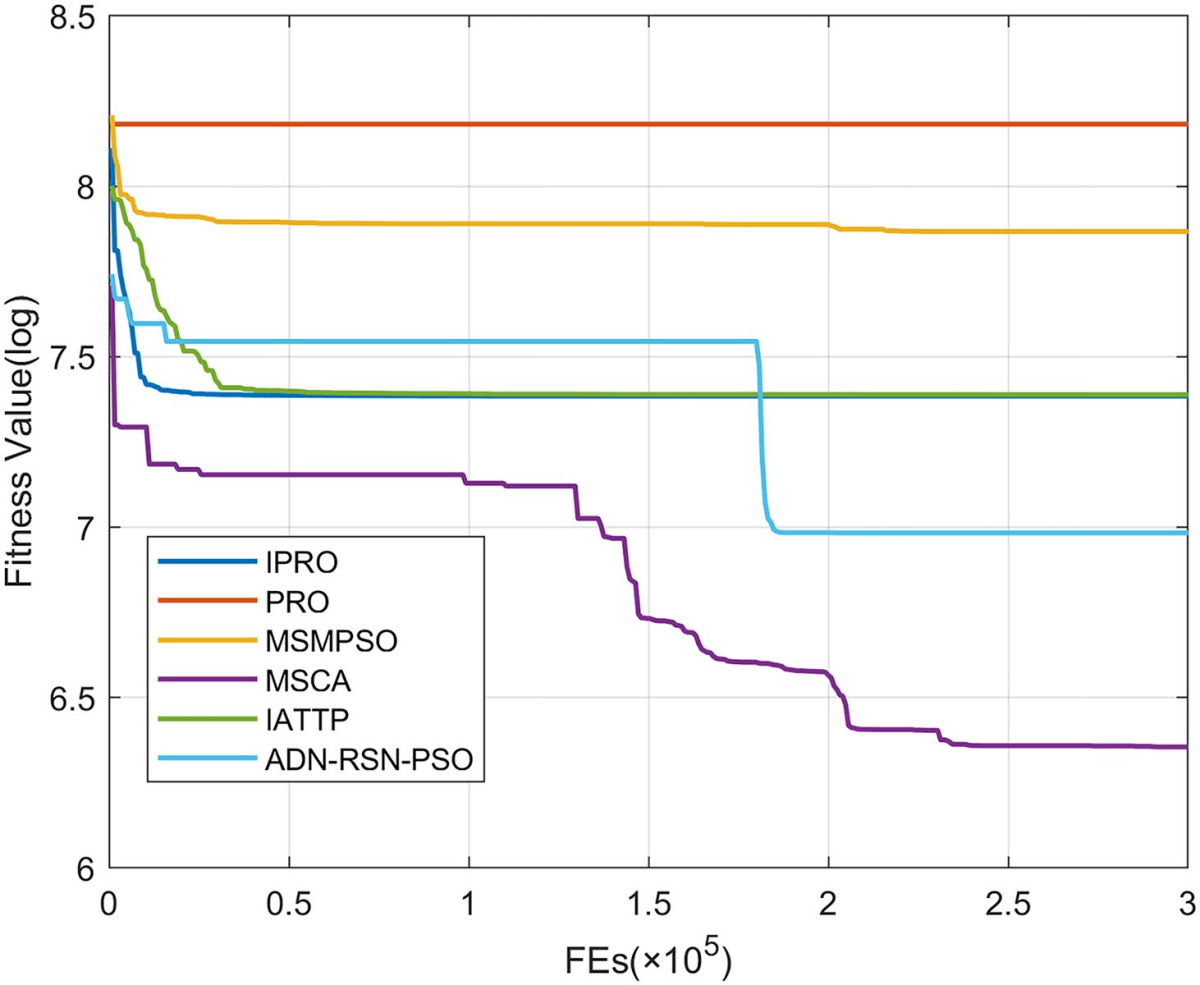
Convergence curves on F27.

**Fig 28:**
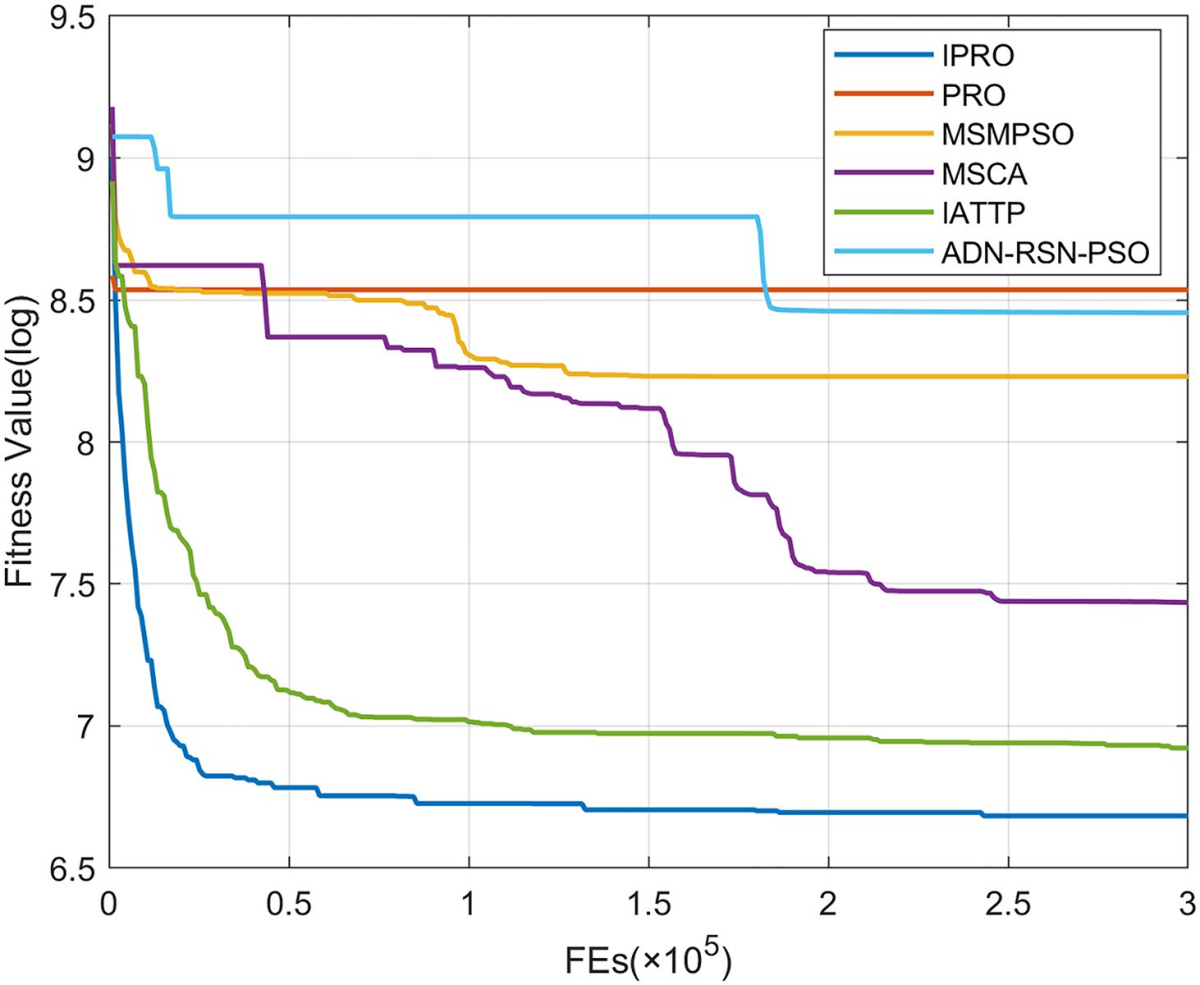
Convergence curves on F28.

Figs 1–28 shows that, for the unimodal functions F1, F2, F3, and F5, the IPRO shows better convergence compared with the other algorithms. For F1, IPRO rapidly converges to the global optimum, whereas the other algorithms fall into the local optimum. For F3, only PRO falls into the local optimum, whereas the other algorithms rapidly converge to the global optimum. Specifically, IPRO, IATTP, and MSMPSO rapidly converge to the global optimum at the initial stage. For F2, F4, and F5, all algorithms fall into the local optimum. For F4, our IPRO shows a better convergence compared with the other algorithms, yet slower than IATTP. Among 15 multimodal functions(including F6 to F20), our IPRO quickly obtains the global optimum of six functions (including F7, F9, F11, F12, F13, and F20) at the beginning. For functions F7, F9, F12, F13, and F20, our IPRO shows better convergence compared with the other algorithms. For F11, our IPRO shows slower convergence than MSCA, yet more quickly than the other algorithms. For F6, F10, F18, and F19, similar to the other algorithms, our IPRO falls into the local optimum, but shows a fastest convergence speed. For F8, our IPRO shows better converagence compared with PRO, MSMPSO, MSCA, and AND-RSN-PSO at the initial stage, whereas IPRO only shows a better convergence than PRO in the end. For F14, all algorithms fall into the local optimum in the early stage of convergence, including IPRO, PRO, MSMPSO, MSCA, IATTP, and AND-RSN-PSO, whereas only AND-RSN-PSO escapes the local optimum in the end. For F15, F16, and F17, our IPRO shows a fastest convergence speed at the initial stage. As evolution goes on, for F15 and F16, each algorithm fall into the local optimum, and AND-RSN-PSO obtain a highest precision solutions. For F17, AND-RSN-PSO escapes the local optimum. For the composition functions (including F21, F22, F23, F24, F25, F26, F27, and F28), each algorithms shows a rapid convergence in the initial stage, but falls into the local optimum in the end. For F21, F24, and F28, IPRO shows a fastest convergence speed and the best quality solutions. For F23, F25, and F26, IPRO only shows a lower quality solution than one algorithm, i.e. AND-RSN-PSO, IATTP, and MSCA, respectively. For F22 and F27, IPRO shows worse convergence compared with MSCA and AND-RSN-PSO. In sum up, IPRO performs well in terms of convergence speed compared with the other four algorithms.

In sum, compared with the other four algorithms, IPRO showed advantages in terms of convergence accuracy and convergence speed. In addition, our proposed IPRO achieved the global optimal of three out of five unimodal functions, and obtained the global optimal of six out of fifteen multimodal functions. Which indicates that, compared with the composition functions, our IPRO is very effective for the unimodal functions and multimodal functions.

## 5. Conclusions

This paper designed the IPRO algorithm to further improve the convergence speed and accuracy of the recently developed population-based algorithm PRO. IPRO differs from PRO in three ways. First, a different approach was used to divide the population into the poor and rich sub-population. At the early stage of convergence, those individuals with the better fitness values were included in the rich sub-population, whereas all the others were included in the poor sub-population. This process resulted in a rich sub-population was larger than the poor sub-population. However, at the final stage of convergence, the poor sub-population was larger than the rich sub-population. Second, the individual updating mechanism of the rich was strengthened, by allowing each individual in the rich sub-population to learn from the poor and the best individuals, instead of form the best individual in the poor sub-population. This procedure increased the convergence speed of IPRO and minimized losses in swarm diversity. Third, the individual updating mechanism of the poor was strengthened based on Gauss mutation, crossover strategy, and new evolution strategy, which maintain the swarm diversity to some extent. The performance of the IPRO algorithm on 28 benchmark functions was verified, compared with that of four the-state- of- the-art meta-heuristic optimization algorithms using the CEC2013 test suite.

Given its employment of new search strategies, IPRO has a slightly higher time complexity than the original PRO algorithm. Therefore, the subsequent work will aim to reduce the time complexity and improve optimization efficiency of the IPRO. Future research on the proposed IPRO may explore its application in solving other real-word problems to further validate its flexibility in generating optimum solutions to a wide variety of optimization problems.
